# Correction: Environmental adversity is associated with lower investment in collective actions

**DOI:** 10.1371/journal.pone.0246591

**Published:** 2021-01-29

**Authors:** N. Lettinga, P. O. Jacquet, J-B. André, N. Baumard, C. Chevallier

The fourth author's name is spelled incorrectly. The correct name is: N. Baumard. The correct citation is: Lettinga N, Jacquet PO, André J-B, Baumard N, Chevallier C (2020) Environmental adversity is associated with lower investment in collective actions. PLoS ONE 15(7): e0236715. https://doi.org/10.1371/journal.pone.0236715

There are errors in the Funding statement. The correct Funding statement is as follows: This study was supported by the EUR FrontCog grant ANR-17-EURE-0017, the Institut national de la santé et de la recherche médicale (INSERM), an ERC consolidator grant (no. 8657) and an “Action Incitative” funding from the École Normale Supérieure. The funders had no role in study design, data collection and analysis, decision to publish, or preparation of the manuscript.
