# Supplementary material for: Environmental adversity is associated with lower investment in collective actions
Source: PLoS One. 2020 Jul 30;15(7):e0236715. doi: 10.1371/journal.pone.0236715 (PMC7392252; doi:10.1371/journal.pone.0236715)
Supplement: S6 Table — (DOCX) [file pone.0236715.s006.docx]

# S6 Table. World Values Survey collective action items.

| Volunteering |
| --- |
| Unpaid work social welfare service for elderly, handicapped or deprived people |
| Unpaid work religious or church |
| Unpaid work education, arts, music or cultural activities |
| Unpaid work labour unions |
| Unpaid work political parties or groups |
| Unpaid work local political action groups |
| Unpaid work human rights |
| Unpaid work environment, conservation, animal rights |
| Unpaid work professional associations |
| Unpaid work youth work |
| Unpaid work sports or recreation |
| Unpaid work women´s group |
| Unpaid work peace movement |
| Unpaid work organization concerned with health |
| Political action |
| Signing a petition |
| Joining in boycotts |
| Attending lawful/peaceful demonstrations |
| Joining unofficial strikes |
