# Supplementary material for: Environmental adversity is associated with lower investment in collective actions
Source: PLoS One. 2020 Jul 30;15(7):e0236715. doi: 10.1371/journal.pone.0236715 (PMC7392252; doi:10.1371/journal.pone.0236715)
Supplement: S1 Text — (DOCX) [file pone.0236715.s008.docx]

# S1 Text. European Values Study discovery sample – results.

The scaled CFI value (0.886), the scaled RMSEA value (0.042) and the scaled SRMR value (0.017) are fairly consistent with a close-fitting model. Therefore, the approximate fit indices reveal no strong misspecification for this model.

| Model part | Latent | Indicator | unstd.c | se | z | p-val | ci.lower | ci.upper | std.c |
| --- | --- | --- | --- | --- | --- | --- | --- | --- | --- |
| Measurement model | Childhood adversity | Parental education | 1.00 | - | - | - | 1.00 | 1.00 | 0.54 |
|  |  | Parents problems making ends meet | 0.36 | 0.17 | 2.15 | 0.03 | 0.03 | 0.68 | 0.11 |
|  |  | Parents problems replacing things | 1.87 | 0.21 | 8.80 | <0.001 | 1.45 | 2.29 | 0.60 |
|  |  | Death of father | 1.44 | 0.63 | 2.28 | 0.02 | 0.20 | 2.68 | 0.09 |
|  |  | Death of mother | 2.13 | 1.03 | 2.07 | 0.04 | 0.12 | 4.15 | 0.07 |
|  | Reproduction-maintenance | Health | -0.34 | 0.01 | -23.41 | <0.001 | -0.37 | -0.31 | -0.38 |
|  |  | Age at 1st birth | -1.61 | 0.07 | -22.94 | <0.001 | -1.75 | -1.48 | -0.33 |
|  |  | Number of children | -0.01 | 0.01 | -0.39 | 0.70 | -0.03 | 0.02 | -0.01 |
|  | Collective action | Volunteering | 0.37 | 0.02 | 17.54 | <0.001 | 0.33 | 0.41 | 0.55 |
|  |  | Political action | 0.41 | 0.03 | 15.79 | <0.001 | 0.36 | 0.46 | 0.60 |
| Structural model | Reproduction-maintenance | Childhood adversity | 0.09 | 0.01 | 10.94 | <0.001 | 0.07 | 0.10 | 0.30 |
|  | Collective action | Reproduction-maintenance | -1.00 | 0.09 | -10.77 | <0.001 | -1.19 | -0.82 | -0.71 |
|  | Collective action | Childhood adversity | -0.04 | 0.01 | -4.19 | <0.001 | -0.06 | -0.02 | -0.10 |
| Covariance | Age at 1st birth | Number of children | -1.11 | 0.04 | -28.61 | <0.001 | -1.18 | -1.03 | -0.22 |

**Supplementary table S1:** European Values Study discovery sample – results

A significant part of the effect of childhood environmental adversity on adult involvement in collective action is mediated by the reproduction-maintenance trade-off (indirect effect: UnStd c = -0.0023 (0.003), bootstrapped ci lower = -0.0029, bootstrapped ci upper = -0.0016, *z* = -6.81, *p* < 0.001).
