# Supplementary material for: Environmental adversity is associated with lower investment in collective actions
Source: PLoS One. 2020 Jul 30;15(7):e0236715. doi: 10.1371/journal.pone.0236715 (PMC7392252; doi:10.1371/journal.pone.0236715)
Supplement: S3 Text — (DOCX) [file pone.0236715.s010.docx]

# S3 Text. European Values Study listwise deletions - mediation effects.

When the reproduction-maintenance trade-off is excluded from the model the effect of childhood environmental adversity on collective action is significant (UnStd c = -0.08 (0.01), *z* = -10.60, *p* < 0.001, Std c = -0.25). When we include the reproduction-maintenance trade-off in the model the following effects are found:

- Indirect effect (UnStd c = -0.08 (0.01), *z* = -7.00, *p* < 0.001, Std c = -0.21)
- Direct effect (UnStd c = -0.03 (0.01), *z* = -3.50, *p* < 0.001, Std c = -0.08)
- Total effect (UnStd c = -0.11 (0.01), *z* = -9.55, *p* < 0.001, Std c = -0.30)

Thus, the effect of childhood environmental adversity on collective action is reduced from -0.25 to -0.08 but is still significant after including the mediator reproduction-maintenance-trade-off. This indicates a partial mediation.
