# Supplementary material for: Environmental adversity is associated with lower investment in collective actions
Source: PLoS One. 2020 Jul 30;15(7):e0236715. doi: 10.1371/journal.pone.0236715 (PMC7392252; doi:10.1371/journal.pone.0236715)
Supplement: S4 Text — (DOCX) [file pone.0236715.s011.docx]

# S4 Text. World Values Survey discovery sample – results.

The scaled CFI value (0.967), the scaled RMSEA value (0.045) and the scaled SRMR value (0.017) are consistent with a close-fitting model. Therefore, the approximate fit indices reveal no strong misspecification for this model.

| Model part | Latent | Indicator | unstd.c | se | z | p-val | ci.lower | ci.upper | std.c |
| --- | --- | --- | --- | --- | --- | --- | --- | --- | --- |
| Measurement model | Reproduction-maintenance | Health | -0.20 | 0.03 | -6.93 | <0.001 | -0.25 | -0.14 | -0.29 |
|  |  | Number of children | 0.26 | 0.04 | 6.75 | <0.001 | 0.18 | 0.33 | 0.19 |
|  | Collective action | Volunteering | 0.22 | 0.03 | 7.67 | <0.001 | 0.16 | 0.27 | 0.14 |
|  |  | Political action | 0.55 | 0.10 | 5.31 | <0.001 | 0.35 | 0.75 | 0.69 |
| Structural model | Reproduction-maintenance | Current adversity | 0.34 | 0.05 | 6.82 | <0.001 | 0.24 | 0.44 | 0.62 |
|  | Collective action | Reproduction-maintenance | -0.56 | 0.16 | -3.53 | <0.001 | -0.87 | -0.25 | -0.61 |
|  | Collective action | Current adversity | 0.08 | 0.06 | 1.25 | 0.21 | -0.04 | 0.20 | 0.16 |

**Supplementary table S1:** World Values Survey discovery sample – results

The effect of current environmental adversity on adult involvement in collective action is mediated by the reproduction-maintenance trade-off (indirect effect: UnStd c = -0.005 (0.001), bootstrapped ci lower = -0.007, bootstrapped ci upper = -0.003, *z* = -4.90, *p* < 0.001).
