# Supplementary material for: Environmental adversity is associated with lower investment in collective actions
Source: PLoS One. 2020 Jul 30;15(7):e0236715. doi: 10.1371/journal.pone.0236715 (PMC7392252; doi:10.1371/journal.pone.0236715)
Supplement: S5 Text — (DOCX) [file pone.0236715.s012.docx]

# S5 Text. World Values Survey imputed data – results.

## Analyses

For the imputed data twenty complete datasets were generated by fully conditional specifications for categorical and continuous data using the R package *mice* [1]. Different imputation methods were used depending on the type of missing data. Predictive mean matching was used for numeric indicators, logistic regression imputation for binary data and proportional odds model for ordered categorical indicators with more than two levels. The function *runMI* of the R package *semTools* [2] as used to combine the results obtained for the 20 imputed datasets.

For the mediation analyses the bootstrap method developed by Preacher & Hayes [3] was used, which is recommended by MacKinnon et al. [4]. This is a non-parametric resampling test. The main feature of this test is that it does not rely on the assumption of normality. Bootstrapping estimates the upper limit and the lower limit of the confidence intervals of an indirect effect. We computed bootstrapped 95% confidence intervals (1000 bootstrap samples) for each of the 20 imputed datasets and then took the average of these datasets.

## Model fit

The scaled CFI value (0.957), the scaled RMSEA value (0.049) and the scaled SRMR value (0.018) are consistent with a close-fitting model. Therefore, the approximate fit indices reveal no strong misspecification for this model.

## Measurement model

The standardized regression weights can be found in S1 Fig. “Subjective health status” (UnStd c = -0.14 (0.04), *z* = -3.90, *p* < 0.001, Std c = -0.24) and “number of children” (UnStd c = 0.20 (0.05), *z* = 3.90, *p* < 0.001, Std c = 0.16) loaded significantly on reproduction-maintenance trade-off. The pattern of covariation follows our predictions: poorer reported health and higher number of children. Hence, the reproduction-maintenance trade-off is consistent with prior studies [5,6].

“Volunteering” (UnStd c = 0.20 (0.03), *z* = 5.96, *p* < 0.001, Std c = 0.13) and “political action” (UnStd c = 0.56 (0.14), *z* = 3.91, *p* < 0.001, Std c = 0.71) loaded significantly on the collective action latent variable, whose greater values indicate higher investments in political activities and, to a lesser extent, higher investments in volunteering activities.

**
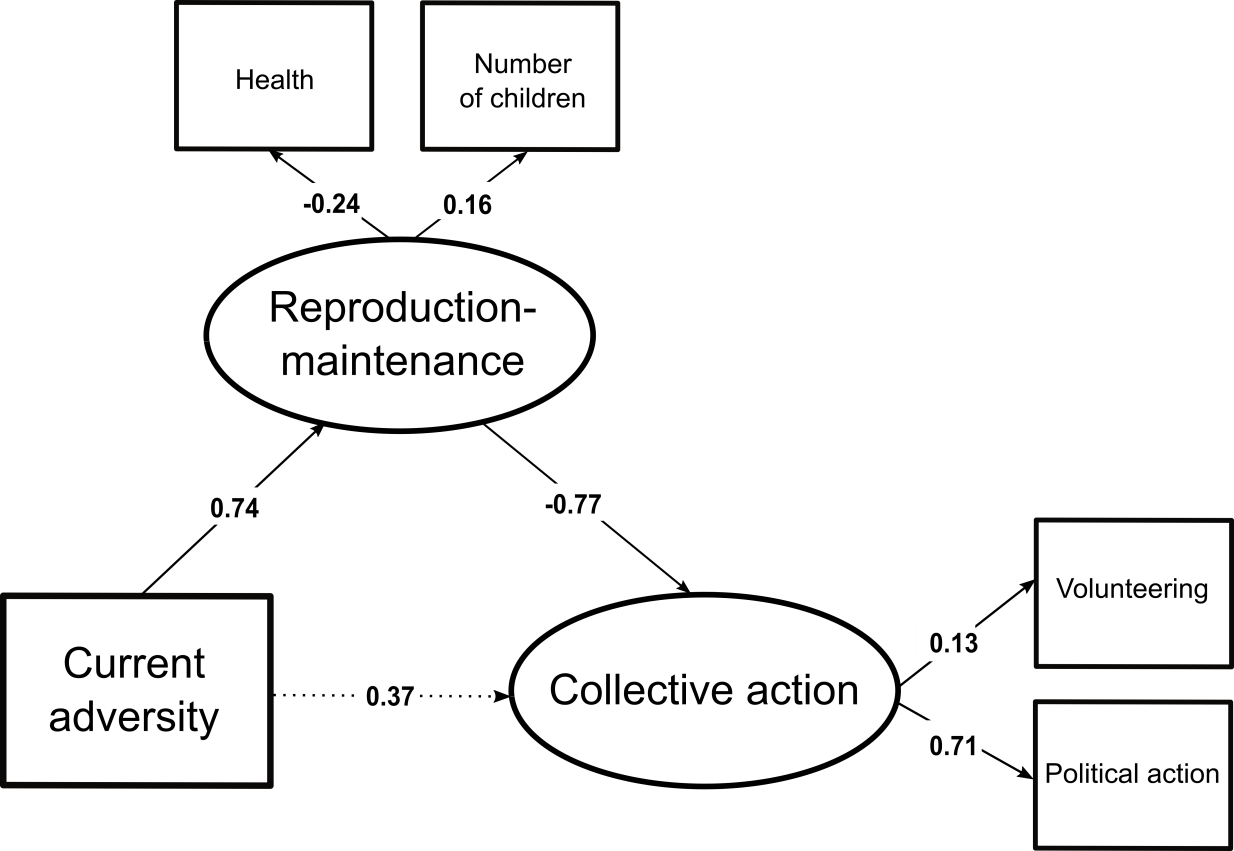
**

**S1 Fig. World Values Survey standardized parameter values estimated by the structural equation model.** Significant paths at the 5% level are represented with a continuous arrow.

## Structural model

S1 Fig shows that the direct effect of current adversity on adult involvement in collective action is not significant (UnStd c = 0.19 (0.18), *z* = 1.05, *p* = 0.30, Std c = 0.37). Furthermore, more current adversity is associated with variations in individuals’ reproduction-maintenance trade-off (UnStd c = 0.47 (0.12), *z* = 3.83, *p* < 0.001, Std c = 0.74), specifically an increased investment in reproduction and a decreased investment in somatic maintenance. The reproduction-maintenance-trade-off is itself associated with lower adult involvement in collective action (UnStd c = -0.62 (0.29), *z* = -2.18, *p* < 0.05, Std c = -0.77). In line with our second hypothesis, the effect of current adversity on adult involvement in collective action is mediated by the reproduction-maintenance trade-off (indirect effect: UnStd c = -0.003 (0.001), bootstrapped ci lower = -0.005, bootstrapped ci upper = -0.001, *z* = -3.28, *p* < 0.001).

## References

1. Buuren SV, Groothuis-Oudshoorn K. mice: Multivariate imputation by chained equations in R. Journal of statistical software. 2010:1-68. doi: 10.18637/jss.v045.i03.

2. Contributors S. SemTools: Useful tools for structural equation modelling. R Package Version 0.4-14. 2016.

3. Preacher KJ, Hayes AF. Asymptotic and resampling strategies for assessing and comparing indirect effects in multiple mediator models. Behav Res Methods. 2008 Aug 1;40(3):879–91. doi: 10.3758/BRM.40.3.879.

4. MacKinnon DP, Lockwood CM, Williams J. Confidence limits for the indirect effect: Distribution of the product and resampling methods. Multivar Behav Res. 2004 Jan 1;39(1):99–128. doi: 10.1207/s15327906mbr3901_4.

5. Brumbach BH, Figueredo AJ, Ellis BJ. Effects of harsh and unpredictable environments in adolescence on development of life history strategies. Hum Nat. 2009;20(1):25–51. doi: 10.1007/s12110-009-9059-3.

6. Mell H, Safra L, Algan Y, Baumard N, Chevallier C. Childhood environmental harshness predicts coordinated health and reproductive strategies: A cross-sectional study of a nationally representative sample from France. Evol Hum Behav. 2018 Jan 1;39(1):1–8. doi: 10.1016/j.evolhumbehav.2017.08.006.
