# Supplementary material for: Environmental adversity is associated with lower investment in collective actions
Source: PLoS One. 2020 Jul 30;15(7):e0236715. doi: 10.1371/journal.pone.0236715 (PMC7392252; doi:10.1371/journal.pone.0236715)
Supplement: S6 Text — (DOCX) [file pone.0236715.s013.docx]

# S6 Text. World Values Survey listwise deletions - mediation effects.

When the reproduction-maintenance trade-off is excluded from the model the effect of current adversity on collective action is significant (UnStd c = -0.11 (0.01), *z* = -8.00, *p* < 0.001, Std c = -0.25). When we include the reproduction-maintenance trade-off in the model the following effects are found:

- Indirect effect (UnStd c = -0.20 (0.12), *z* = -1.73, *p* = 0.08, Std c = -0.42)
- Direct effect (UnStd c = 0.12 (0.11), *z* = 1.12, *p* = 0.26, Std c = 0.25)
- Total effect (UnStd c = -0.09 (0.02), *z* = -3.89, *p* < 0.001, Std c = -0.18)

Thus, the effect of current adversity on collective action is changed from -0.25 to 0.25 but turns non-significant after including the mediator reproduction-maintenance trade-off. This indicates a full mediation.
