# Supplementary material for: Environmental adversity is associated with lower investment in collective actions
Source: PLoS One. 2020 Jul 30;15(7):e0236715. doi: 10.1371/journal.pone.0236715 (PMC7392252; doi:10.1371/journal.pone.0236715)
Supplement: Attachment — Submitted filename: Lettinga et al 2020-to authors.pdf [file pone.0236715.s014.pdf]

Environmental harshness is associated with lower investment in collective actions

Lettinga, N., Jacquet, P.O., André, J-B., Baumard, N., Chevallier, C.

Reviewer: John Lazarus

Limitations of review. I am not expert in structural equation modelling and I do not have experience working with either of the data sets used here.

General comments

1. This is a valuable study in a currently lively and somewhat controversial area of research. It is particularly useful in measuring associations of prosociality with both environmental harshness and measures predicted by life history strategy (LHS) theory.

2. The paper tests the hypotheses that prosociality is reduced in adults both in a harsher current environment and when raised in a harsher environment during childhood. The paper is clearly written, the Introduction covers the relevant literature well and the Discussion section is commendable for the way it considers limitations of the study; problems of causal inference from correlational data; and alternative evolutionary explanations for the findings.

3. Throughout the paper the authors describe various behavioural measures examined by others, and by themselves in this paper, as ‘cooperation’. In evolutionary biology cooperation is defined as a social action resulting in a benefit to both (all) parties in the interaction. The behaviours analysed here are volunteering and political action, behaviours that would be defined in evolutionary biology as ‘altruism’; that is, actions resulting in a benefit to the recipient(s) and a cost to the self, and the authors agree that these two behaviours do have this cost-benefit structure (lines 187, 189-90 and 475 [later numerical references are also to line numbers]). Similarly some behavioural measures in previous studies cited here as cooperative (e.g. reference 30) are actually altruistic (e.g. volunteering and charitable giving).

Since this paper is testing hypotheses within the discipline of evolutionary human studies it is important that the terminology is correct. This is far from being a dry terminological matter; rather it is central to the hypotheses tested here, as I shall go on to argue. The authors are testing the idea that in harsh

environments there is a 'need to favour immediate over long-term benefits' (54) and they state that 'cooperation is a future-oriented strategy: in the short term, it is more advantageous to reap immediate, smaller, but more certain benefits by cooperating less' (62-64).

I have a number of comments related to this point:

- (a) The behavioural measures employed here – volunteering and political action – are altruistic not cooperative as defined above. Consequently they are suitable for testing the hypothesis that they will be less evident in harsh environments, but this is because they are immediately costly, and not, as argued in the paper, because benefits from them are delayed.
- (b) I do not agree with the claim quoted above (62-64, also 19-20) that cooperation is necessarily a 'future-oriented strategy'. It is future-oriented in the case of reciprocal altruism where, after an exchange of altruistic acts between two individuals, both have gained a net benefit (but is only future-oriented if the focal individual is the first altruist). (Volunteering and political action, in themselves, do not fall into this category – rather they are altruistic – although one can imagine scenarios in which they are followed by a reciprocal benefit.) But where the cooperation involves joint simultaneous activity there is an immediate benefit to both parties (compared to not cooperating).
- (c) In consequence of this last point, and following the arguments of the authors, simultaneous cooperation, as defined here, would be predicted to be more common under harsher conditions since it provides an immediate benefit in comparison with the decision to refrain from cooperation.
- (d) In using a 'proxy' measure for cooperation (e.g. 95-6) it is necessary that the proxy satisfies the definition of cooperation given above. I do not mean here the distinction between behavioural and attitudinal measures (472-3), which is, of course, important. Rather, I mean that whether it is a behavioural or an attitudinal measure it should satisfy the above definition of cooperation (which for an attitude would refer to an imagined cost-benefit structure).
- (e) I suggest that the authors look again at their use of the terms 'cooperation' and 'altruism', and their derivatives, in light of these comments. At the same time it would be useful also to check use of the terms 'prosociality' and 'collective action'. 'Prosociality' refers to a social action providing a benefit to the recipient and therefore includes both altruism and cooperation, as defined above. I would like to see a

definition in the paper of 'collective action' since it is used a number of times. My next point argues, more generally, for the importance of clear definitions and distinctions between the different types of prosocial behaviour for this field of research.

4. Following on from comment (e), it seems to me that the discrepant findings in the literature about the effect of environmental harshness on prosociality (the authors refer to this at 42-6) may be partly a function of the literature not distinguishing carefully enough between different types of social interaction, with potentially different cost-benefit structures for the players; broadly cooperation versus altruism.

I declare an interest here. I have a paper (Lazarus 2017, and included with this review), one not cited by the authors, in which I review evidence from humans and other taxa that cooperation (defined, as above, in evolutionary biology) may be an inverted-U function of environmental adversity (i.e. harshness). The review is accompanied by an explanatory evolutionary model that differs from the ideas discussed in the present paper (i.e. selection for present-focussed decision making in harsh conditions). The model is intended to supplement, and not to replace, explanations based on present-future trade-offs. The authors may wish to refer to my paper.

My paper also discusses the point made in the paragraph before last and I quote the passage here (Lazarus 2017, section 6, p. 590):

6. Other forms of prosociality
Cooperative behaviour shares motivational and emotional features, and evolutionary and cultural origins, with related prosocial behaviours, beliefs and attitudes, and the influence of adversity has been studied here too. At the level of individual differences those with more adverse life experience exhibit more empathy, compassion and generosity in charitable giving and aid to a stranger (Lim and DeSteno 2016; Lim 2017). The influence of social class on prosociality is controversial, with recent conflicting findings for a range of measures: utilitarianism, empathy, feelings of entitlement, narcissism, theft, lying, cheating, helpfulness, generosity, trust, trustworthiness, volunteering and charitable donation. Some studies find greater prosociality in higher classes (Korndörfer, Egloff, and Schmukle 2015) but others find the opposite class effect (Côté, House, and Willer 2015), to mention just two of the most recent reports. In understanding these apparently conflicting findings it will be helpful to develop more tailored predictions for each measure, as attempted here for

cooperation. If the inverted-U relationship proposed here between environmental quality and cooperation should hold also for some of these other forms of prosociality this might resolve some of the (apparent) inconsistencies in the data, since conflicting studies could be situated on opposite sides of the environmental inflection point. As others have noted it is also important to take account of the methodology by which these results have been obtained, from behavioural observations and experiments to self-report, in both the lab and the real world, since they each have their own psychological influences.

I invite the authors to engage with these ideas.

Detailed comments

Title: Since the LHS variables play a major role in the association between environmental harshness and the prosociality variables I suggest that LHS is added to the title.

76. 'By contrast' with the previous sentence these references are expected to deal with the effect of the experience of safe conditions during human childhood. However, two seem not to be relevant: [54] is a comparative mammalian paper and [60] is a study of guppies.

121 and 338. Provide references or web links to the two data sets.

123. The countries in the survey: it would be useful, in the supplementary material, to provide lists of the countries in each study uniquely, and in both studies. Country differences may help to explain some differences in the results of the two studies, and will also be useful for comparison with future studies.

134. 'in' should read 'into'.

135-7 & 370-72. 'In both subsamples, we excluded participants with too many empty cells (more than two standard deviations from their respective subsample mean)'. I find this unclear; 'empty cells' seems to refer to missing data whereas the passage in parentheses seems to refer to outliers.

151. 'parent's' should read 'parents'. 'end' should read 'ends', both here and in the figures.

166. This should probably read ‘health costs are induced’.

177. ‘no’ should read ‘not’.

248 and 403. Discovery and replication samples in the two studies. In the Results section of each study there is analysis of just one set of data. It is not stated whether this is the discovery sample, the replication sample, or the two combined. If it is one of the two samples then the second should be added, or comparison made with the first. If it is the two combined then any differences between the two samples should be described.

255-9. Additional analysis with imputed data, to deal with the problem of missing responses. ‘The results of this analysis are consistent with the results obtained using listwise deletions (see S1 Text in the Supporting Information)’ (257-9). In contrast to this statement in the paper there is an important difference in the results of the two analyses. In the main text analysis the authors conclude from a significant negative association between childhood harshness and collective action (309 and Figure 2) that ‘This implies that a harsher childhood environment is associated with less investment in collective actions later in life’ (309-10). However, the same conclusion is drawn from the additional analysis although now the same correlation is significant and positive (S1 Text, p. 3, lines 3-4 and Figure S1). This requires Discussion in the main text, particularly since it represents a core prediction of the paper.

282-7 and S1 Text. Discussion of the effect of the remaining independent variable, parental education, is missing. Is this because of the standardisation procedure?

291. There seem to be words missing at the end of this line.

29. State what the ‘expected way’ is.

303 & 444. ‘bold arrow’ would probably be better as ‘continuous arrow’, in contrast to the dotted arrows. Certainly in the typescript one cannot tell bold from normal lines.

364-7. Excluding respondents without children. Further explanation would be useful here since the variable ‘only relevant for respondents with children’ (365-6), age at first birth, is not used in this second study (but is in the first). So

were childless respondents excluded so that the two studies would be more similar in their respondent characteristics?

390. Insert ‘respectively’ after ‘study 1’?

480. ‘whether people cooperate less under harsher conditions . . .’?

517. ‘administrating’ should read ‘administering’.

531. Delete ‘shocks’?

537-9. Either ‘correlates’ should read ‘correlated’ if the correlation was within the study or a reference should be given if it was a correlation with another study.

560 ‘Their model’ would read better as ‘The model’.

830. Add journal name.

Reference

Lazarus, J. (2017) Cooperation in adversity: An evolutionary approach. *Global Discourse* 7 (4): 571–598, doi: 10.1080/23269995.2017.1402426
