# Supplementary material for: Environmental adversity is associated with lower investment in collective actions
Source: PLoS One. 2020 Jul 30;15(7):e0236715. doi: 10.1371/journal.pone.0236715 (PMC7392252; doi:10.1371/journal.pone.0236715)
Supplement: Attachment — Submitted filename: 2017 Lazarus Cooperation in adversity an evolutionary approach.pdf [file pone.0236715.s015.pdf]

Global Discourse
An Interdisciplinary Journal of Current Affairs and Applied Contemporary Thought

ISSN: 2326-9995 (Print) 2043-7897 (Online) Journal homepage: http://www.tandfonline.com/loi/rgld20

Cooperation in adversity: an evolutionary approach

John Lazarus

To cite this article: John Lazarus (2017) Cooperation in adversity: an evolutionary approach, Global Discourse, 7:4, 571-598, DOI: 10.1080/23269995.2017.1402426

To link to this article: https://doi.org/10.1080/23269995.2017.1402426

Published online: 02 Jan 2018.

Submit your article to this journal

View related articles

View Crossmark data

Citing articles: 2 View citing articles

Full Terms & Conditions of access and use can be found at
http://www.tandfonline.com/action/journalInformation?journalCode=rgld20

Download by: [Dr John Lazarus] **Date:** 02 January 2018, At: 13:16

GLOBAL DISCOURSE, 2017
VOL. 7, NO. 4, 571–598
https://doi.org/10.1080/23269995.2017.1402426

Routledge
Taylor & Francis Group

RESEARCH ARTICLE

Check for updates

Cooperation in adversity: an evolutionary approach

John Lazarus

Centre for Behaviour and Evolution, Institute of Neuroscience, Henry Wellcome Building, Newcastle University, Newcastle upon Tyne, UK

ABSTRACT

Throughout the organic world cooperation provides mutual benefit but is vulnerable to exploitation from free-riders. Over the last 30 years work in evolutionary biology and game theory has provided understanding of the conditions necessary for the maintenance of cooperation, and advances in gene-culture coevolution theory have extended this understanding to our own species. After a preamble on the evolutionary analysis of behaviour I outline this work. I then consider how cooperation is influenced by environmental adversity and find that in non-human species it is enhanced under these circumstances in a range of taxa. In a sample of human cases the same result is found in a majority, but the opposite effect in some when socioeconomic position is the measure of quality. In anthropological studies of societies living *in extremis*, again the opposite effect is found. I propose a sigmoid shape for the relationship between adversity and fitness (or human well-being) and a consequent inverted-U shaped relationship between adversity and the benefit of cooperation. Most of the data presented on the relationship between adversity and cooperation are consistent with this proposal. I suggest further tests of the proposal and place the study of cooperation in the broader context of prosociality.

ARTICLE HISTORY
Received 1 September 2017
Accepted 6 November 2017

KEYWORDS
Adversity; austerity; cooperation; disadvantage; harshness; precarity; prosociality; risk

Downloaded by [Dr John Lazarus] at 13:16 02 January 2018

1. Introduction

Cooperation offers benefits through the sharing of physical power, resources, skills, knowledge, problem-solving experience, social support and social influence. But cooperation is a fragile condition, vulnerable to exploitation from those who take without contributing. Many disciplines have important contributions to make to its study (Lazarus 2003) and here I apply an evolutionary approach (and for humans a gene-culture coevolutionary approach; see Section 3) to understand what happens to cooperation – throughout the organic world but focusing on our own species – in conditions of adversity. Does it flourish or wither, and why?

Cooperation, as understood in biology and economics, is defined in terms of its consequences for those involved in a social interaction and this is the approach I take here. To be precise an act is cooperative if it results in a benefit to both the actor and the recipient(s) of the act. My metric for evaluating benefit in non-human species is, in principle, Darwinian fitness, although empirically we often have to make do with a

CONTACT John Lazarus j.lazarus@ncl.ac.uk Centre for Behaviour and Evolution, Institute of Neuroscience, Henry Wellcome Building, Newcastle University, Newcastle upon Tyne, UK

© 2017 Informa UK Limited, trading as Taylor & Francis Group

proxy for fitness. By Darwinian fitness I mean lifetime reproductive success, the total number of offspring produced. In the human case, where a gene-culture coevolutionary approach is more appropriate, we need to be more cautious in assuming that choices are always adaptive in a Darwinian sense, and I explain this more fully in Section 2.

Defining adversity

An environment is defined as adverse if it has some negative impact on the species concerned. For an evolutionary analysis, and for non-human species, the metric for this impact is fitness. For our own species it is also often the case that fitness is lower in what we deem to be a more adverse environment. However, as I have already said, we cannot assume that all human behaviour is adaptive and it is more parsimonious to claim simply that what I will call 'well-being' is reduced in a more adverse environment, my term being simply a more concise equivalent of 'subjective well-being' which combines measures of cognition (satisfaction) and positive affect (Cummins 2000). I discuss the relationship between well-being and fitness further in Section 2.

Broadly, adversities have abiotic or biotic origins. In the first category are variables such as temperature, aridity and altitude for which a species will have some optimal value at which its fitness (or well-being) is greatest, larger departures from this value bringing greater adversity. Biotic adversity arises from predators, parasites, disease and competitors. Uniquely human adversities, both abiotic and biotic, include pollution, housing, employment, health services, education, poverty, lack of opportunity and the social environment: other people who individually, collectively or institutionally may harm another physically, emotionally, economically or in any other way, either actively or by withholding some good. Adversity may also be felt in terms of relative deprivation (Davis 1959; Wilkinson and Pickett 2010).

Adversity (or harshness as it is also termed) clearly does impact on human well-being and consequently has profound and widespread influences on cognition, behaviour and development beyond its impact on cooperation (Low 1990; Ellis et al. 2009; Frankenhuis, Panchanathan, and Nettle 2016). These influences arise from sources that range from the abiotic environment through the personal to the societal. For example, lower socio-economic status is associated with a 'behavioural constellation of deprivation' that leads to a focus on present-oriented behaviours (Pepper and Nettle 2017). Further, some aspects of adversity, such as extrinsic mortality risks (Nettle 2010; Frankenhuis, Panchanathan, and Nettle 2016; Pepper and Nettle 2017) and economic and political factors (Standing 2011), are outside the individual's control.

Outline

Following a preamble on evolutionary explanation (Section 2) I describe current thinking on the evolutionary origin of cooperation in the human species (Section 3). Then, following an account of how cooperation is influenced by adversity (Section 4) in organisms generally, but focusing on the human species, I offer a contribution to the explanation of these relationships (Section 5). Finally, I extend the discussion briefly to other forms of prosociality (Section 6) and draw conclusions (Section 7).

Downloaded by [Dr John Lazarus] at 13:16 02 January 2018

Downloaded by [Dr John Lazarus] at 13:16 02 January 2018

2. A preamble on the evolutionary analysis of behaviour

Given their very different intellectual histories, social and evolutionary scientists have largely worked independently to understand human behaviour and social structures, and when they have interacted it has been more often in conflict than in productive dialogue. Matters have improved as evolutionary biologists have come to appreciate that any evolved behavioural predisposition must emerge as action through the processes of individual development occurring within a particular cultural environment. And having learned these general lessons from psychology, anthropology and sociology they have gone on to formalize the ways in which organic and cultural evolution interact, as discussed below. But since the rapprochement is not yet complete, it will be useful, I think, to outline the nature of the questions the evolutionary scientist asks about behaviour and the conceptual approach that is brought to bear in searching for answers, before proposing some ideas for understanding cooperation under adversity that have an evolutionary-cultural foundation.

The social scientist seeking a causal understanding of human action is concerned with what the biologist calls *proximate causation*. That is, finding the influences within the environment (including the social and cultural environment), and within the individual, that account for the behaviour of interest influences that the psychologist calls motivational. And, by extension, an understanding is sought for how behaviour varies between individuals and cultures. The evolutionary behavioural analyst asks, in the first place, a different question, one that the biologist calls *ultimate causation*: what were the evolutionary forces (generally forces of natural selection) that have resulted in behaviour appearing in the particular form that it does, in response to particular proximate influences? And the guiding principle in generating hypotheses about ultimate causation is that it is predicted – under the influence of natural selection – to produce *adaptive* behaviour: behaviour that efficiently solves a problem in the organism's life.

But there is a second stage of evolutionary analysis; hypotheses of ultimate causation lead naturally to complementary hypotheses about the environmental cues – the proximate causes – predicted to influence the emergence of behaviour patterns given their proposed adaptive function (Barkow, Cosmides, and Tooby 1992). This is clearly a different source for ideas about proximate causation to those from the social sciences, but one that has a strong foundation in the theory of natural selection.

The generation of hypotheses of proximate causation from natural selection theory has been a particular feature of evolutionary psychology (Tooby and Cosmides 1990) and the evolutionary logic underlying this approach is important for the ideas I present in Section 5 concerning the influence of adversity on cooperation. Using this relationship as an exemplar the argument from evolutionary psychology is that natural selection over our evolutionary history has been responsible for the learned psychological predispositions that we bring to cooperative decision-making in the contemporary world, as well as the proximate causes that turn these predispositions into actions. I would argue that this is a particularly strong premise for our present case since cooperative decisions, environmental adversity and the interaction between the two must have had a great impact on fitness throughout human history. These predispositions govern contemporary behaviour to the extent that 'present conditions resemble past conditions in specific ways made developmentally and functionally important by the design of those

adaptations' (Tooby and Cosmides 1990, 375). The approach is perfectly compatible with the evidence that cooperative tendencies vary with the economic and societal structures of different cultures, variation which can sometimes also be understood in adaptive terms (Henrich et al. 2004) as a result of further learned predispositions. Whether any of these predispositions are optimal in terms of fitness enhancement in the changed environments of the modern world remains an open question, is not assumed by the evolutionary psychology approach and is not assumed here. And since the consequences of cooperative decisions for people are my primary focus I will, parsimoniously, refer to 'well-being' (rather than fitness) to describe the relative positive outcomes of cooperating or not cooperating in environments of differing qualities. In the non-human examples the outcome measures are either fitness or, more frequently, proxies for fitness.

An example of how the evolutionary psychology approach seeks to understand evolved proximate causes will be useful here. Lieberman, Tooby, and Cosmides (2003) tested Westermarck's (1921) theory for the proximate causation of incest avoidance, an adaptive phenomenon in that it reduces the damaging effects of inbreeding depression. Westermarck proposed that incest avoidance, and the moral objection to incest, were achieved by the co-residence of siblings from an early age resulting in 'sexual negative imprinting'. Lieberman and coworkers found support for their hypothesis, derived directly from Westermarck's proposal, that duration of co-residence with an opposite-sex sibling would correlate positively with the strength of the moral opposition to sibling incest. The association was independent of degree of relatedness (adopted, step-, half- or full-sib) while relatedness itself, the *functionally* important factor, did not influence the moral attitude to incest. This is understandable in evolutionary terms since a child cannot reliably know its kin relationship to another child it grows up with. Duration of co-residence, however, is a reliable cue since experienced directly, and crucially it correlated significantly with relatedness, the functionally relevant variable. These results illustrate the point that evolved proximate causal factors need not themselves represent the adaptive variable but must map reliably onto it.

Evolutionary biologists and social scientists continue to generate their hypotheses concerning proximate causes from different principles. This is a difficult division to bridge, but on another area of dispute further mutual understanding should be possible. This is the role of genetics in the causation of behaviour and, in particular, the worry by some social scientists that an evolutionary analysis of behaviour assumes genetic determinism, the notion that a particular genetic make-up fully determines a behaviour; given gene X behaviour Y will be shown and will be shown whatever the environment throws at it. While some biologists may have held this view in the past, it is now a straw man. The notion of innateness is discredited; rather behaviour is understood to unfold during life as a continuing interaction between the individual, its genotype and the environment (Mameli and Bateson 2006; Bateson and Mameli 2007), including the cultural environment (Nettle 2009).

It is the complex interplay between the individual, its genotype and the environment just described that is subject to natural selection, behaviour responding flexibly and often adaptively to the environment through processes involving direct experience, social influence and the internalization of norms. Although this means that behaviour is subject to various biases and that we are not blank slates (Pinker 2002), it does not

Downloaded by [Dr John Lazarus] at 13:16 02 January 2018

necessitate genetic determinism, just as an enlightened view of the power of the environment to influence behaviour does not merit it with an analogous determinism.

Further, there is no longer a fundamental conflict between the study of culture and of biological evolution as forces for change. In the discipline known as 'gene-culture coevolution' the two are now integrated (Boyd and Richerson 1985; Richerson and Boyd 2005). The logic of the approach is that cultural practices modify the human environment and consequently influence the selection pressures acting on the human genome and directly on the cultural practices themselves, producing feedback loops, both positive and negative. Cultural transmission may be horizontal, vertical or oblique, and natural selection is assumed to act on both genetic and cultural variation in behaviour and cognition, although cultural success may look very different to genetic success. Culture evolves and the methods of evolutionary biology can be used to study its evolution. And gene-culture coevolution theory now has a new importance following recent findings that cultural practices modifying the environment have resulted in changes in gene frequencies (Laland, Odling-Smee, and Myles 2010; Richerson, Boyd, and Henrich 2010). It is therefore no longer possible to dismiss the idea that cultural forces might have changed the human genome by claiming that there has not been sufficient time for natural selection to act. And indeed natural selection continues to act on the human genome (e.g. Byars et al. 2010).

3. How did cooperation evolve in our species?

Understanding the evolutionary origin of cooperation has been a challenge since helping others would not at first sight appear to be favoured by natural selection. The method required to analyse this problem is game theory, developed by mathematicians and economists to predict rational choices when two or more individuals interact and the choices they make influence the payoff for other players. Economic rationality is classically deemed to be self-regarding in that it maximizes some kind of personal payoff (Gintis 2003). However, achieving maximum payoff may not be possible when the consequence of one's choices is under the influence of the choices made by others, as in a social interaction. Instead of reaching the 'best' choice, defined by maximum payoff, therefore, rational players in a game come to settle on the set of choices which means that *no player can do better by choosing to play differently*, such a set of plays being termed a *Nash equilibrium* (Binmore 2007a, 2007b; Colman 1999).

Evolutionary biologists took this economic equilibrium concept in games and applied it to a similar problem, in which natural selection determines the decisions made by the rational player. If at the Nash equilibrium no player can make a more profitable move this is just the outcome to be expected when individual decisions are evolving under the force of natural selection, with fitness as the payoff metric. And, following this logic, the evolutionary equivalent of a Nash equilibrium is termed an *evolutionarily stable strategy*, or *ESS* (Maynard Smith 1982). Both the Nash equilibrium and the ESS are stable equilibrium states and thus, by definition, are what we expect to see in nature. An important difference between the two concepts, however, is that while the Nash equilibrium must take rational play as an *assumption*, a state of affairs on which players cannot improve is *built into* the theory of natural selection and therefore also the ESS concept.

Downloaded by [Dr John Lazarus] at 13:16 02 January 2018

Downloaded by [Dr John Lazarus] at 13:16 02 January 2018

The game theory approach to understanding the conditions for the existence of cooperation can be exemplified by the well-known economic game, the prisoners' dilemma (e.g. Colman 1999). In this game two individuals interact in a scenario in which each actor has a choice of two plays or strategies which, in general terms, can be thought of as cooperating with (C) or defecting on (D) the other player. The original prisoner scenario is unnecessarily complicated and I will illustrate the dilemma with a simpler scenario described by Colman (1999). A Buyer has decided to purchase a diamond from a Seller and a price has been agreed. For some reason the exchange must be made in secret and so the two agree each to leave a bag, the Buyer's containing the agreed price and the Seller's the diamond, at a different place in a wood, after which each will retrieve the other's bag. The problem is, of course, that either party might be tempted to leave an empty bag, thus defecting (D) on the other, rather than cooperating (C) with a full bag. Figure 1 shows the relative payoffs to Buyer and Seller of the four possible outcomes of the exchange. The absolute value of the numbers in the figure is arbitrary; all that matters, and what defines the dilemma, is their ranking, a higher number indicating a more preferred outcome.

If both parties cooperate and fulfil their agreement (a CC outcome), they gain three points, but if both come with an empty bag (DD), they gain only two since they have both failed to close a deal they desired. If one party leaves an empty bag (D) and the other a full one (C), then the defector goes home with both the cash and the diamond (five points), while the other party, the sucker, has neither (0 points).

What is the equilibrium outcome to this game, represented by the best response of each player to the play of the other? The answer, which can be seen in Figure 1, is for both players to defect (DD) since *whatever the other player does it is always more profitable to defect than to cooperate*. The dilemma demonstrated by the game is that this outcome, though a result of rational play, is not the best outcome that can be achieved. Both parties would clearly prefer a CC outcome to a DD outcome, but even if there was some way for them to agree on such an outcome, it would still pay to renege on the agreement.

This game captures the essence of the problem of how to maintain cooperation and the logic can be simply extended to interactions between more than two players. Why should a hunter exert himself fully in the hunt, and why should he share his catch with others in his hunter–gatherer group? Why pull your weight in a team effort or, as a

		Buyer	
		Cooperate (Bag full)	Defect (Bag empty)
Seller	Cooperate (Bag full)	3, 3	0, 5
	Defect (Bag empty)	5, 0	2, 2

Figure 1. Payoff matrix for the prisoners' dilemma. The first payoff in each cell is to the seller.

nation, fulfil promises on reducing greenhouse gas emissions? The temptation to defect is often rational in economic (selfish) terms.

It would therefore seem that an ultimate explanation of the fact that cooperation *is* a feature of human social life cannot rest on the logic of the prisoners' dilemma as I have described it, in which the two parties meet only once. In the real world we often enter into relationships in which we interact repeatedly over days, months or years, and an ultimate explanation must take into account the fact that for most of our evolutionary history we lived in small groups in which all identities were mutually known and people interacted repeatedly throughout their lives (Kelly 2013). In the language of game theory we played iterated (i.e. repeated), not one-shot, games with each other and thus had the opportunity to reward past support, punish past defections or break off a relationship altogether. This complicates enormously the strategies that can be played, compared to the one-shot game I have described, strategies that take into account the history of the relationship. In particular, the fear of retaliation in later encounters encourages cooperation and can be the basis for a cooperative ESS in the repeated prisoners' dilemma, such as the Tit-for-Tat strategy: start by cooperating, then copy partner's last play (Axelrod and Hamilton 1981; see also Nowak and Sigmund 1993; for another cooperative ESS). And, pre-empting these findings, the 'folk theorem', as game theorists call it, concluded that for indefinitely repeated games with little discounting of future payoffs cooperative equilibrium strategies will always exist (Binmore 2005).

So cooperation can result from self-regarding, broadly reciprocal, interactions between pairs of individuals (so called *direct reciprocity*). But understanding how relationships develop in small communities is not just a matter of summing all the dyadic relationships within it. Individuals learn about the cooperativeness of others by interacting with them, observing them directly and talking to third parties (Dunbar 2004). In this way they build up *reputational* knowledge invaluable when responding to offers of interaction with the potential for mutual benefit, or when selecting partners for such interactions themselves (Roberts 1998; Gurven 2004; Craik 2009). Alexander (1987) was the first evolutionary biologist to emphasize the importance of this process for the evolution of cooperation. He coined the term *indirect reciprocity* to describe the biasing of cooperative responses to those known to have been cooperative to others in the past, and there is now experimental evidence that players are more likely to cooperate with other cooperators than with free-riders in small group interactions (e.g. Milinski et al. 2001; Barclay 2004).

The analysis so far shows that self-regarding rationality is compatible with cooperation when individuals interact repeatedly with known partners. However, laboratory and real-world experiments show that people are not fully selfishly rational since participants also cooperate in one-shot prisoners' dilemma games about half the time and contribute in one-shot public goods games, the multi-person equivalent of the prisoners' dilemma in which the rational decision is to give nothing (Camerer 2003). Behaviour in another and much simpler game, the dictator game, is particularly instructive. In this game a dictator simply decides how to split an amount of money between themselves and another player. The rational self-regarding choice is clearly to give nothing away, but giving 10–20% of the fund is common (Camerer 2003). The results from this and other games played in many different societies while showing much variation – understandable in terms of the economic and societal structure of the culture – demonstrate that

Downloaded by [Dr John Lazarus] at 13:16 02 January 2018

Downloaded by [Dr John Lazarus] at 13:16 02 January 2018

pure selfishness is rare and suggest the existence of what may be a universal sense of fairness (Henrich et al. 2004).

Such a predisposition for fairness may have been favoured by selection, acting both genetically and culturally (Chudek and Henrich 2011), since it undermines self-regarding rationality and eases the path to the more rewarding cooperative CC (rather than DD) outcome in prisoners' dilemma-type repeated encounters and makes it possible in one-shot encounters too. In a manner that is similar to Roberts's (2005) notion of interdependence, one way of representing the fairness motive is by adding some proportion of the other player's payoff to one's own. If this proportion is great enough (e.g. >0.67 for the payoffs in Figure 1) the Nash equilibrium and ESS become CC even in the one-shot game.

Binmore (2005, Chapter 9) argues that our sense of fairness evolved as a stable social mechanism for sharing resources in the non-hierarchical societies of the earliest hunter–gatherer humans and that we carry this same sense today. If we extrapolate from our knowledge of present-day egalitarian hunter-gatherers, our earliest human ancestors benefited from sharing with their neighbours because of their interdependence (Roberts 2005) – particularly in cooperative hunting and gathering, and food sharing – and consequently had a stake in each other's well-being. In particular, such cooperative practices reduce the risk of periods without food, and free riding on this system is not tolerated (Winterhalder 1986; Kaplan, Hill, and Hurtado 1990; Gurven 2004; Kelly 2013, Chapters 6, 7). (It is probably not a coincidence that the cooperative non-hierarchical structure, collective decision-making, monitoring of resource acquisition by others, graded sanctions for defectors and conflict resolution mechanisms of many hunter–gatherer societies (Kelly 2013) are all features shared with successful common pool resource groups such as coastal fisheries and forestry systems (Ostrom 1990).) An early human social contract characterized by sharing, as a form of enlightened self-interest, may be the origin of the Golden Rule – variations on 'Do as you would be done by' – probably the most universal ethical imperative that we have (Binmore 2005, Chapter 9).

While an early evolutionary origin for our widespread sense of fairness seems likely, scholars differ on how best to explain the fact that we often act fairly in *one-shot* interactions in the real contemporary world (e.g. queuing or returning a lost wallet) and in the lab, when reciprocity is not expected. One explanation is that people think in such situations, consciously or not, *as if* they were repeated non-anonymous games, since the predisposition we bring to such encounters is one that evolved in the small groups of our early evolutionary history described above (e.g. Haselton and Nettle 2006). Gintis et al. (2003) disagree, arguing that early humans would also have engaged in encounters with a low probability of continuing, in which defection would have been the more profitable strategy. However, this doesn't explain the *cooperative* responses that are regularly seen in one-off encounters in both the real world and the laboratory. A further point is that experimental one-shot interactions may be played like repeated games since they inadvertently share cues associated with the reputational indirect reciprocity consequences of being observed by others (Kurzban 2001; Haley and Fessler 2005; Bateson, Nettle, and Roberts 2006).

The behavioural expression of cooperation, or its absence, is inevitably accompanied by emotions including a feeling for the welfare of others, guilt, shame, a personal concern for reputation and a fear of punishment (Milinski et al. 2001; Fehr and Gachter 2002; Barclay 2004; Bowles and Gintis 2011), and develops in the individual

under the influence of social norms (e.g. Krupka and Weber 2013; Hugh-Jones and Ooi 2017). It is possible to incorporate these processes into models that combine biological and cultural evolution, as discussed in the previous section, and such a model, simulating internalization of norms transmitted vertically between generations and obliquely by socialization institutions, concludes that cooperation can be maintained when a minority of the population exhibit *strong reciprocity*, cooperating unconditionally and punishing defectors at a personal cost (Gintis 2003). Although the importance of strong reciprocity for the evolution of cooperation is currently a matter of controversy (Guala 2012), the model exemplifies an approach to the problem of understanding *how* individuals and communities reach equilibrium prosocial states. This must occur by some combination of genetic evolution, cultural influence and direct experience, but we are some way from a full understanding of the processes involved and their interaction.

Some discussion of altruism is also necessary here since, although formally distinguished from cooperation, the two prosocial acts share the consequence of benefitting others, differing in that the actor also benefits from a cooperative act but suffers a cost from an altruistic act. It is important to note that focusing narrowly on a single act defined, as above, as altruistic may miss a bigger picture. The single act may be just one of a series of reciprocal altruistic exchanges, so that considered over a longer time period the relationship is seen to be one of cooperation, as in reciprocal food sharing, since both parties benefit. Here the success of cooperation relies on trusting that the other party will reciprocate. In the dictator game described above the single decision involved is 'purely' altruistic if anything is given to the other party, as it commonly is. Acts of cooperation and altruism, in addition to their direct consequences, may accrue delayed benefits due to direct and indirect reciprocity and may be selected for as honest signals of the ability to act in this way in the future (Roberts 1998).

However, a difference between cooperation and altruism important for us here is that acts of pure altruism favoured by selection in the small societies of our early history, due to delayed benefits, may not be personally beneficial in the large anonymous societies of the contemporary world, even though they continue to be selected by gene-culture coevolution due to internalization of norms built on evolved predispositions. The upshot of this, as I will go on to explain, is that *I deal here with the influence of adversity on cooperation only* and not on pure altruism as just described. This is partly because I am concerned with the more straightforward case where costs and benefits are borne in a given individual (the cooperative actor) *in a given environment* in the here and now. In pure altruism immediate benefit is borne only by another party who may inhabit a different (social) environment from the altruistic actor, such as the case of Christians who rescued Jews in Nazi-occupied Poland (Tec 1986) and those who rescued persecuted family members, friends and strangers in Argentina during the military rule of 1976–1983 (Casiro 2006). Any delayed benefits to the self of altruism through direct or indirect reciprocity, if there are any at all, take place in a future environment of an uncertain nature. In addition, even if one wanted to assume that our altruistic tendencies are built on the evolved predispositions mentioned above, it would be very difficult to quantify the contribution of supposed delayed benefits to our altruistic decision-making. For all these reasons altruism is not amenable to the analysis I describe in Section 5. My analysis is suitable for examining cooperation, however, for which I need to consider only direct costs and benefits to the self and not to others (although even

Downloaded by [Dr John Lazarus] at 13:16 02 January 2018

here the analysis is not perfect since cooperation may also have delayed benefits for the self).

This account of the origins of human cooperation just scratches the surface of half a century of research on the topic (Ridley 1997; Hammerstein 2003; Gintis et al. 2005; Tomasello 2009; Bowles and Gintis 2011). My aim here has been to outline the kinds of thinking required for an understanding of the origin and maintenance of human cooperation as a prelude to the following discussion of the influence of adversity on cooperative behaviour.

Finally, we should not exaggerate the human tendency to cooperate; like all human traits it is variable and some individuals, in some situations, prefer to freeride on the generosity of others.

4. Adversity and cooperation: data

What follows is by no means an exhaustive or systematic review but I have not been selective. I report all my findings from the literature on the influence of adversity on cooperation.

Non-human cases

There is a widespread tendency in the natural world for organisms to be more cooperative in conditions of adversity and I have not located any evidence to the contrary. The phenomenon has been reviewed by Andras and Lazarus (2005) and many of the following examples are taken from that account:

- In response to environmental stressors, individual bacteria become social and form multi-cellular structures such as biofilms and mushroom bodies (Greenberg 2003) which enhance their resistance to the stressor, such as an antibiotic (Drenkard and Ausubel 2002).
- Social, in contrast to solitary, feeding in the nematode *Caenorhabditis elegans* is triggered by environmental stressors (De Bono et al. 2002).
- Fish school and primate group sizes are larger where predation risk is greater (Seghers 1974; Farr 1975; Hill and Lee 1998). Gregariousness reduces predation risk in various ways (Krause and Ruxton 2002).
- Colonies of the common mole-rat (*Cryptomys hottentotus hottentotus*) are larger in arid areas, which present greater foraging adversity, than in mesic (moderately moist) areas. Movement between colonies is also less frequent in arid areas. Larger and more stable colonies favour resource sharing and the development of cooperative relationships with known individuals (Spinks, Jarvis, and Bennett 2000).
- The phenomenon is also found in plant communities, in which an individual plant, acting respectively competitively or cooperatively, can inhibit or promote the biomass, growth and reproduction of its neighbours. In 11 mountain habitats around the world relationships between neighbours in subalpine plant communities are more competitive, whereas in the corresponding alpine communities, where abiotic stress is higher, cooperative interactions predominate (Callaway et al. 2002).

Downloaded by [Dr John Lazarus] at 13:16 02 January 2018

The human case

For the human case I have sought data from a range of methodologies: real-life case studies and experiments; anthropological work; within- and between-society comparisons; self-report measures; and lab experiments. For reasons that will become clear in Section 5 I divide the data into those in which, like the non-human data, cooperation increases with adversity, followed by those in which the opposite is the case.

Cooperation increases with adversity

While there is a long-standing view in the social sciences that external threats increase group cohesion (Stein 1976), this is not always measured in behavioural terms. However, it seems to be commonplace that people caught up in a natural disaster cooperate in ways they would not consider under more normal circumstances. Members of the Committee on Disaster Studies of the National Academy of Sciences, USA, write that following a natural disaster:

> The net result … is a dramatic *increase* in social solidarity among the affected populace … The sharing of a common threat to survival and the common suffering produced by the disaster tend to produce *a breakdown of pre-existing social distinctions* and a great outpouring of love, generosity, and altruism … persons tend to act toward one another spontaneously, sympathetically, and sentimentally, on the basis of common human needs rather than in terms of predisaster differences in social and economic status. (Fritz and Williams 1957, 48, emphasis added)

This account provides something of a control condition in making a comparison with pre-disaster behaviour.

In the trench warfare of World War I cooperation between British and German infantry was commonplace. Between battles a 'live and let live' reciprocal arrangement developed whereby both sides refrained from firing on the enemy, in spite of the wishes of their commanders. This peaceful arrangement could be signalled, for example, by repeated daily firing at precisely the same position at precisely the same time. Axelrod (1984; drawing on Ashworth 1980) analyses this as an iterated prisoners' dilemma in which, for the infantrymen at least, mutual cooperation was the best outcome.

These real-world case studies show that *some* people behave cooperatively under severe adversity, sometimes at great personal cost, but they do not allow a quantitative comparison with the frequency or degree of cooperative behaviour in less adverse conditions. To provide the kind of data we are seeking here systematic studies are required.

The set of studies probably closest to these real-world cases of external threat are the experiments, carried out mostly in the lab (e.g. Puurtinen and Mappes 2009; Puurtinen, Heap, and Mappes 2015), but also in the real world (Erev, Bornstein, and Galili 1993) in which participants in small groups are found to cooperate more (generally through donations in a public goods game) when competing with other groups. The adversity in these studies is inferred to arise from the competition; it is intrinsic to the task and not a pre-existing condition that participants bring to the experiment, as in the studies that follow. While this is an important distinction, for present purposes it needs to be examined in relation to my analysis in the following section, which is framed in terms of the individual's perception of their adversity. A

Downloaded by [Dr John Lazarus] at 13:16 02 January 2018

Downloaded by [Dr John Lazarus] at 13:16 02 January 2018

temporary adverse stimulus that arises immediately before a decision has to be made may have different consequences from that of a long-term adverse condition that one brings to an experiment, such as might arise from low social status or hunger, say. However, there are many psychological studies in which brief priming stimuli are remarkably effective in imitating the influence of long-term conditions, including those of prosociality (Piff et al. 2010; Piff 2014; Nettle et al. 2014). Since brief exposure to competition in an experiment may also have such a priming effect, it seems worthwhile to consider competition experiments as potentially suitable for our analysis here.

Moving on to an anthropological study, in examining the influence of adversity using a random half of the societies in the standard cross-cultural sample, Low (1990) found a significant positive association between extreme cold and the hunting of large game, which is a cooperative enterprise.

Finally, in a US study of social class effects, with class measured by educational attainment and income, lower-class participants offered more points than upper-class participants in a trust game, a cooperative game involving trust that the partner will reciprocate (Piff et al. 2010).

Cooperation decreases with adversity

The results of the following two studies conflict with those of Piff et al. (2010) just described and I will return in the following section to the issue of how these results might be resolved.

In his study of Tyneside neighbourhoods Nettle (Nettle 2015; Nettle, Colléony, and Cockerill 2011; Schroeder, Pepper, and Nettle 2014) has compared 'the informal social relationships and interactions that make up so much of everyday life' (Nettle 2015, 12) of two neighbourhoods in the city of Newcastle upon Tyne, UK. These neighbourhoods, with populations of about 3000 each, are markedly different in adversity as measured by the Index of Multiple Deprivation. The more deprived neighbourhood is at the first percentile of deprivation in England, while the less deprived is at the 79th percentile, and the study was carried out at 'a moment when people in [the former] neighbourhood had endured many years of uncertainty about the future of the whole area' (12). A number of behavioural and self-report measures were made, but the only ones relevant here were two self-report measures; adult respondents in the less-deprived neighbourhood trusted each other more (in two studies) and felt more strongly that people in their neighbourhood looked out for one another. The finding on trust was replicated for children between 9 and 15 years of age across a range of neighbourhoods differing in the level of deprivation. (Trust is not the same as cooperation but it is required if cooperation through repeated altruistic exchanges is to be successful.)

In support of these findings Haushofer (2013) found, in large data sets from the *World Values Survey*, that trust increased significantly with income within countries and with per capita GDP (at purchasing power parity) across countries.

Finally, I come to the tail of the distribution of environmental harshness, where case studies are naturally rare and where experiments are not possible. I describe three cases of societies living in conditions of extreme adversity; societies *in extremis*.

Burch (2006, 272), as described by Kelly (2013, 288n6), found that in Alaskan Iñupiaq Eskimos ‘in periods of widespread famine and hunger, the [cooperative] distribution system broke down, families hoarded food, and some tried to steal the stores of others or even to kill the owners’.

In 1969 and 1970 Laughlin (1974, 1978) studied the So, a small society in Northern Uganda: ‘The total So ecological/economic picture is grim. It is one of progressive deterioration of alternative resource bases to the point where a period of drought brings extreme and widespread hardship and starvation to the people of So’ (Laughlin 1974, 380).

Laughlin quantified ‘generalized’ reciprocal exchange in the So, ‘where the emphasis is upon the act of exchange and not upon immediate return or making a profit’ (Laughlin 1974, 381) and compared its expression in two study periods, ‘one of extreme hardship [and a second] in relationship to the first a time of plenty’ (385). He found more generalized reciprocity in the period of plenty and, although the data are not analysed statistically, the increases compared to the period of extreme hardship are quite large: 48% for feeding guests (‘the major medium of generalized reciprocity’ [386]), 124% for food transfers and 38% for total (food + non-food) transfers. Generalized reciprocity contrasts with ‘negative reciprocity’ (i.e. exchange for profit) as a means of resource acquisition and when one type of reciprocity declines it is generally compensated by an increase in the other. This reminds us that in the real world – in contrast to the lab – cooperation is just one means of getting on in the world and is therefore subject, indirectly, to a range of influences, some of which may also be responsive to adversity. Thus in the So, to compensate for the reduction in generalized reciprocity during hardship, the cash value of negative reciprocity increased in that period by 454%.

The So are related to the Ik, who live further north in the mountains of northern Uganda. When studied by Turnbull (1966, 119–136, 1967, [1972] 1974, 1978) from 1964 to 1966, it would seem that the Ik lived in even greater hardship than the So, and their society certainly suffered from a greater absence of cooperativeness. The Ik’s former nomadic hunting cycle had been curtailed by the government and this, together with the frequent droughts, led to intermittent famine and eventually the ‘disintegration of Ik society’ (Turnbull 1978, 53), Turnbull’s period of fieldwork coming at ‘a critical moment in the process of social change’ (Turnbull 1978, 53), though 5 years after Turnbull’s study, Joseph Towles, Turnbull’s collaborator, found the social system much the same (Turnbull 1975, 355). In Turnbull’s account cooperation was limited to house building, which required more than a single builder, and individuals passed their lives in relative isolation. Family and community bonds of care broke down completely, children were weaned at the age of three and then left to forage for themselves, and young and old were left to die if they could not find sufficient food. Although children foraged in gangs, these gangs served only as protection against predators (including adult Ik) and food was not shared.

> [A child] learned that cooperation was rarely beneficial – a temporary expediency at best – and that the unpredictability of circumstances that could make it worthwhile meant that there was no value in establishing permanent bonds with others on grounds of age, sex, or kinship. He learned that systematic sociality itself had no value. (Turnbull 1978, 64)

Downloaded by [Dr John Lazarus] at 13:16 02 January 2018

Turnbull concluded that, given the 'extreme … circumstances' (Turnbull [1972] 1974, 111) 'the sadly functional nature of the Ik non-social system … was the only way to survive' (Turnbull 1976, 6).

Turnbull's 1972 book on the Ik, *The Mountain People*, was critically received by some anthropologists, partly for its claimed lack of objectivity (Beidelman 1973; Barth 1974; Wilson et al. 1975; Knight 1976). Two of these eight commentators (Barth and Geddes in Wilson et al. 1975) pointed to inconsistencies in Turnbull's report but none doubted his findings as I describe them above. Heine (1985), who studied the Ik for 2 months in 1983, critiqued many aspects of Turnbull's ethnographic work but again did not dispute the above description of Ik society. The Ik continue to live in northern Uganda today.

5. Understanding the influence of adversity on cooperation

Game theory models have illustrated ways in which environmental adversity selects for cooperation (Andras, Lazarus, and Roberts 2007; Smaldino, Schank, and McElreath 2013). Here I ask more simply, and more generally, *what might the shape of the relationship between adversity and the benefit of cooperation look like*, and *what does that shape allow us to infer about how cooperation will vary with adversity*? These questions were addressed by Andras and Lazarus (2005), with additional mathematical formalities, and I develop the ideas further here. My aim is to see to what extent these proposed relationships might explain the findings described in Section 4. This seems to be a particularly worthwhile exercise given the broad range of contexts and taxonomic groups in which cooperation is enhanced by adversity, while the relationship is reversed *in extremis*. Although I will focus on the human case the conclusions apply to the non-human examples too.

The simple framework to be described is not intended to deny the complexity of environments and of social life but rather to provide an explanation for one feature of that complexity, environmental adversity, while acknowledging that adversity might work on cooperation in additional ways too. Adversity is a ubiquitous variable influencing a wide variety of cooperative interaction types, and it will be achieving its effects alongside a cluster of other influences. My approach does not deny that there are individual differences in what people see as a life of well-being and how that life may be enhanced, or sometimes diminished, by cooperation. As a natural scientist, however, I proceed in the belief that our understanding of how and why people interact with their environment as they do can be advanced by utilizing various methods of behavioural data collection, as well as statistical methods for taking individual differences into account. Finally, I am aware that I have offered only a small sample of human cases and that the pattern of results that emerges does not unequivocally support my analysis. However, studies of these relationships are still in their infancy and a comprehensive understanding is still some way off. In that context the fit between the data and the ideas I present here seems to merit further testing.

It might be thought immediately obvious *why* people seem to cooperate more in adversity, that is, to provide an ultimate, or functional, explanation. People in adversity have a greater 'need', it might be argued, and everything else being equal we would expect that need to be met. Or it might be stated or hypothesized, though without

Downloaded by [Dr John Lazarus] at 13:16 02 January 2018

supporting theory or evidence, that cooperation will be greater in a poorer group (Da Costa, de Melo, and Lopes 2014, 455) or that this relationship has an inverted-U shape (Laughlin and Brady 1978). But as cost-benefit analyses such explanations are incomplete, as I will show. In addition, they may not explain the effect of adversity *in extremis* reported above, which *is* given an explanation in the present analysis.

I start with two general assumptions. First, as already stated, that well-being (and, for non-humans, fitness) declines with adversity or, in other words, that it increases with the *quality of the environment*. Second, where cooperation is observed I assume that on average, and including any delayed benefits, it brings greater well-being than its non-cooperation alternative (but not necessarily for all individuals on all occasions, since there will generally be a dynamic which includes some degree of freeriding). This assumption follows from the game theory prediction of how self-interested individuals will behave when the marginal net benefit of cooperation over non-cooperation (call it *B*) exceeds the threshold value at which cooperation becomes the equilibrium outcome (this kind of analysis was introduced in Section 3 in discussion of the prisoners' dilemma). It follows in turn that cooperation will be more likely to occur where *B* is greater since a greater value of *B* is more likely to exceed the threshold relevant to any particular case. And if *B* increases further, above the threshold value, cooperation is predicted to increase in order to take up this additional benefit, following the same self-interested logic that applied to the switch from non-cooperation to cooperation at the threshold. Such an increase in cooperation can occur by repetition of a cooperative act or by replacing it with a more beneficial alternative, the increase in cooperation being subject to the usual limitations as it comes into conflict with other demands on the individual's time and effort. I consider below the case of how, in fact, *B* is predicted to change in magnitude with environmental quality.

Though it is important to think in terms of net marginal benefit in defining *B*, to take account of potential marginal costs involved in cooperation (i.e. costs involved in cooperating that are absent when not cooperating), in some cases there may be no marginal costs. For example: a hunter or gatherer working with others rather than alone, where the benefit is bringing back more resources per capita; a car-sharing scheme where the parties involved get a ride every day for a share of the overall cost; or a sharing of knowledge or expertise where the gain per capita is enhanced when cooperating.

I represent these two assumptions graphically by two increasing functions relating environmental quality (*E*) to well-being (*W*), one function for the well-being of a cooperating individual and another for non-cooperation, the former function exceeding the latter for all values of *E*, for the reasons just argued. The non-cooperation function does not represent a free-rider's well-being but well-being if cooperation does not occur at all. The 'well-being of a cooperating individual' function represents well-being having provided a given cooperative act (at or above the cooperative threshold), and if this act has a marginal cost over non-cooperation, it is assumed to be independent of the quality of the environment.

Next, I add three further assumptions: that the shape of the increasing relationship between environmental quality and well-being is sigmoid, both when individuals do not cooperate and when they do; and that these two sigmoid functions converge at both extremes (Figure 2). The basis for these assumptions is presented in the Appendix. The

Downloaded by [Dr John Lazarus] at 13:16 02 January 2018

slope of a sigmoid curve at first increases and then decreases; it is first concave upwards (what I shall call the left segment of the curve) and then concave downwards, also called diminishing returns (the right segment). The pair of sigmoid curves, representing cooperation and non-cooperation, stand for the two conditions as they occur within a particular society or other group under analysis; I am not claiming that there is a common scale across all data sets that might be used to test the predictions. And in testing the predictions it will be important to assure, as far as is feasible, that the groups being compared along the environmental quality axis are indeed comparable on all features except that of some measure(s) of environmental adversity.

The marginal net benefit of cooperating (*B*), compared to not cooperating, at a given value of environmental quality (*E*) is therefore the difference in well-being between the cooperation and non-cooperation functions. This is the net benefit of the act over and above the condition of non-cooperation; the benefit gained as a result of the cooperative interaction or enterprise less any marginal cost of the individual's contribution (i.e. cost over and above cost for the case of non-cooperation). It can be readily seen from Figure 2 that *as E increases B at first increases up to an inflection point,* E_i*, on the environmental quality axis and then declines – an inverted-U relationship*. Although any marginal cost of cooperation has been assumed to be independent of environmental quality, this may not hold if resources for cooperation are required in advance of a cooperative relationship being initiated. In this case those in a higher quality environment may find this initial investment more affordable, which would raise the benefit somewhat in those environments.

Since we would expect the occurrence of cooperation to map directly onto its marginal net benefit, *B*, this pattern captures the essence of most of the data I have reviewed, if we assume that the *in extremis* cases lie to the left of E_i, which is very

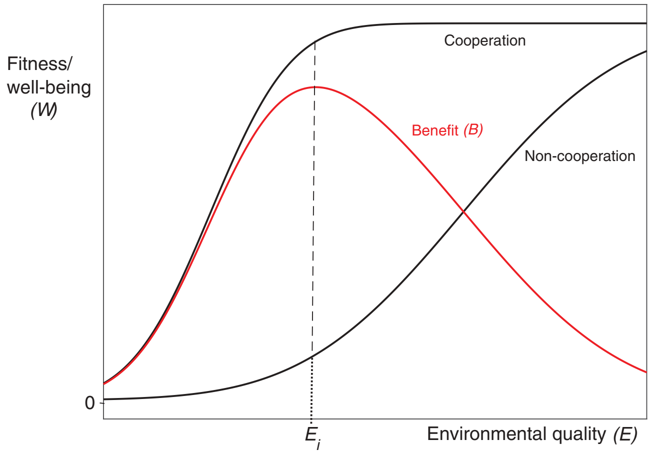

Figure 2. The sigmoid curves are illustrative examples of the proposed relationship between fitness/well-being (*W*) and environmental quality (*E*) in conditions of cooperation and non-cooperation. The inverted-U curve shows benefit (*B*), that is, the difference in fitness/well-being between cooperation and non-cooperation. E_i indicates the inflection point of environmental quality, at which benefit ceases to increase with *E* and begins to decline as *E* increases further. The image is the property of the author.

Downloaded by [Dr John Lazarus] at 13:16 02 January 2018

plausible, and all other cases lie to the right of E_i, which is less certain. That is, cooperation declines with adversity *in extremis* but otherwise the opposite is the case. The sigmoid curves could be drawn in many ways; here I have speculatively drawn them so that E_i sits towards the adverse end of the environmental quality continuum. This is not an a priori assumption that I wish to defend; I have done it simply to capture one *post-hoc* interpretation of the data, which is that the reversal of the 'adversity enhances cooperation' effect is rare and that it mostly occurs in extreme adversity (or in the extreme of *relative* deprivation (Davis 1959; Wilkinson and Pickett (2010)) if relative and not absolute deprivation is the influential variable). A consequence of this position for the inflection point is that as environmental quality worsens beyond E_i the cooperation curve must fall steeply to converge with that for non-cooperation, which means that the benefit of cooperation will also fall steeply. A consequence of this is that below E_i the benefit of cooperation is very sensitive to small changes in environmental quality and I will return to the implications of this.

The cases that do not immediately fit into this scheme are Nettle's (2015) Tyneside neighbourhood study and Haushofer's (2013) multi-national study, in both of which cooperation *declines* with adversity. This divergence in results cannot be resolved with any certainty but I offer some observations. First, the deprived neighbourhood in Nettle's study might lie to the left of E_i, which would mean that it fitted into the present scheme. I do not mean to suggest that this community has a life as harsh as that of the So and the Ik. However, its state of relative deprivation – relative to its UK comparators – may be similar; it is in the 1% of the most deprived neighbourhoods in England. In addition, residents of the neighbourhood may share, but to a lesser extent, the problem responsible for the breakdown of Ik society, that of hunger. A recent account of the relationship between hunger, socioeconomic position (SEP) and behaviour reports that, based on US studies,

> a substantial fraction of people from [low-income] households experience an excess of hunger due to their SEP, at least some of the time [and] … within very affluent populations, individuals of lower SEP eat less satiating diets; do so on more irregular schedules; and a very sizable proportion … report experiences such as food insufficiency and food insecurity that imply an increased frequency of hunger. (cited in Nettle 2017, 7)

Note also that the area containing the deprived neighbourhood contained an emergency-assistance food bank (Nettle 2015, 19). Nettle's (2015) own analysis of his results bears some similarities to the above arguments, including as it does the proposition that the more deprived neighbourhood is 'closer to the edge' (60) in the sense that the only way to avoid a crisis is to cross this edge in the hope of a happy outcome to a risky venture. And when it comes to a potential cooperative interaction:

> [i]f the people in your neighbourhood are … close to the edge, then it makes sense that even if you had a lot of interaction with them you might not feel that you had *enough* interaction to say you knew what they were going to do next. (61, emphasis in the original)

Standing (2011, 20) has a similar conception of the precariat which, he suggests:

> lives with anxiety – chronic insecurity associated not only with teetering on the edge, knowing that one mistake or one piece of bad luck could tip the balance between modest dignity and being a bag lady, but also with a fear of losing what they possess even while feeling cheated by not having more.

Downloaded by [Dr John Lazarus] at 13:16 02 January 2018

My second observation is that the average per capita GDP measure used for Haushofer's (2013) between-country comparison may well be unsuitable for the present analysis since a particular average value may be accompanied by very different distributions of values within the nation, and consequently different values for adversity, depending on how adversity maps to per capita GDP from country to country. Third, it is informative to examine Nettle's and Haushofer's trust measures further. These are the only studies reviewed here that employ self-report rather than behavioural measures and, of course, a trusting attitude is relevant in the present context only to the degree that it predicts cooperative behaviour. While Nettle's measure concerned trust of others within the same neighbourhood, Haushofer's question – 'Do you trust people you meet for the very first time?' – was more generic. This 'generic trust' measure is more problematic as evidence here since it is unclear what the *environment* of any imagined cooperation in the mind of the respondent might be: the respondent's own environment, that of an imagined trustee, or something else? (See Nettle (2015, 63) for parallel comments on social environment.)

My suggestion that Nettle's deprived neighbourhood might lie in the left segment of the sigmoid meets a general problem for an explanation that predicts an inverted U-shaped relationship between two variables. As a specific example of this general problem, for the Tyneside neighbourhoods' case to fit my explanation, the wealthier neighbourhood should not lie so far to the right that it is the *less* cooperative of the two. More generally, with few data points it is difficult to support or refute an inverted U-shaped relationship with confidence. With just two data points, a finding of more cooperation under adversity, less cooperation or no difference in cooperation could all be accommodated in the inverted-U relationship between environmental quality and benefit by placing the data points, respectively, on the right arm of the U, the left arm or one on each arm. However, as more data points become available over a wider range of environmental qualities, it is increasingly possible to support or reject the proposed relationship.

In this context it is important to stress that I am not suggesting that a particular pair of sigmoid curves describes all cases (where by 'cases' I mean studies that compare cooperation between two or more levels of adversity) since different cases will involve different populations and will rarely use the same metrics for adversity and cooperation. To the extent that my thesis is correct, each case of the cooperation–adversity relationship will have its own pair of sigmoid curves, the particular shapes of which (and consequently of the benefit curve) are free to vary. Indeed many cases, as in all the examples described here, will cover too narrow a range of adversities (or will compare too few adversity levels) to reveal a sigmoid relationship and instead the relationship will be monotonic. In this monotonic case my account predicts concave upwards functions below E_i, but concave downwards functions above E_i, additional evidence being required to support a case for one or the other position on the environmental quality axis. Finally, although I argue that all cases cannot be placed *quantitatively* within a single two-dimensional adversity–cooperation space, I do want tentatively to propose, on the basis of the studies I have described here, that Figure 2 may represent a broad and qualitative truth about the adversity–cooperation relationship (cooperation being narrowly and behaviourally defined, as I have stressed). That is, that the relationship is

Downloaded by [Dr John Lazarus] at 13:16 02 January 2018

negative over much of the range of environmental quality but that in the poorest environments it is reversed and is steeper.

To my knowledge there are no data sets showing an inverted-U relationship between adversity and cooperation but this may be because no study (and particularly no experimental study) has considered a broad enough range of adversities. Another strong prediction of my proposition that needs testing is that the *benefit* of cooperation has an inverted U-shaped relationship with environmental quality.

Although environmental quality takes the role here of an independent variable, this does not mean that it is fixed. A cooperative act might have a sufficiently beneficial outcome to move the actor further to the right along the environmental quality axis. Another point about environmental quality, particularly for the human case, is that the best environments may offer opportunities for individuals to find new ways to cooperate, ways that increase the benefit of cooperation and may also improve environmental quality.

This analysis of response to adversity, in which the environmental state is assumed to be fully known, complements that of Haselton and Nettle (2006), based on signal detection theory, for cases in which judgments are made in conditions of incomplete information.

Populations in extremis

I have found no non-human examples where cooperation is reduced by adversity. Under the present proposals, such an example would represent a population to the left of the inflection point and therefore *in* or close to *extremis*. This lack of data could therefore be explained by such populations having a high risk of dying out, migrating to a more suitable environment, or becoming permanently asocial, unless they were fortunate enough to be saved by environmental change or – particularly for human cases – by an outside agency. The risk of extinction or migration would be particularly acute due to the steep decline in cooperative benefit to the left of E_i. Gintis (2003, 160, emphasis in the original) takes a similar view and points to a cruel irony:

> In the primitive conditions under which human sociality evolved, when a group was threatened with extinction or dispersal, say through war, pestilence, or famine, cooperation was most needed for survival. But since the probability that the group will dissolve increases sharply under such conditions, cooperation based on future reciprocation cannot be maintained. Thus, *precisely when the group is most in need of prosocial behavior, cooperation based on repeated interactions will collapse.*

Turnbull (1976, 6) held that, for the Ik, acting alone brought *greater benefits* than cooperating since, to repeat an earlier quote, 'the sadly functional nature of the Ik non-social system … was the only way to survive'. In terms of the present formulation this would mean that the cooperation and non-cooperation curves crossed at the adverse extreme.

It is also possible that acting alone may be more beneficial than cooperating under certain circumstances in the very best environments, the cooperation and non-cooperation curves again crossing (John Baker and Siobhán O'Sullivan, personal communications, 8 January 2017).

Downloaded by [Dr John Lazarus] at 13:16 02 January 2018

Downloaded by [Dr John Lazarus] at 13:16 02 January 2018

6. Other forms of prosociality

Cooperative behaviour shares motivational and emotional features, and evolutionary and cultural origins, with related prosocial behaviours, beliefs and attitudes, and the influence of adversity has been studied here too. At the level of individual differences those with more adverse life experience exhibit more empathy, compassion and generosity in charitable giving and aid to a stranger (Lim and DeSteno 2016; Lim 2017). The influence of social class on prosociality is controversial, with recent conflicting findings for a range of measures: utilitarianism, empathy, feelings of entitlement, narcissism, theft, lying, cheating, helpfulness, generosity, trust, trustworthiness, volunteering and charitable donation. Some studies find greater prosociality in higher classes (Korndörfer, Egloff, and Schmukle 2015) but others find the opposite class effect (Côté, House, and Willer 2015), to mention just two of the most recent reports. In understanding these apparently conflicting findings, it will be helpful to develop more tailored predictions for each measure, as attempted here for cooperation. If the inverted-U relationship proposed here between environmental quality and cooperation should hold also for some of these other forms of prosociality, this might resolve some of the (apparent) inconsistencies in the data, since conflicting studies could be situated on opposite sides of the environmental inflection point. As others have noted it is also important to take account of the methodology by which these results have been obtained, from behavioural observations and experiments to self-report, in both the lab and the real world, since they each have their own psychological influences.

7. Conclusions

Putting aside the case of societies *in extremis* for the moment, I have argued that in a more adverse environment a greater benefit is to be gained by cooperating, and consequently that cooperation will be more common in these circumstances. This view is supported by data from a broad range of non-human species and for a range of human contexts. The possible exception for the human case is the influence of SEP, where recent data show an unresolved picture.

The major premise for my conclusions is that fitness or well-being is a sigmoid function of environmental quality and I have suggested ways in which the implications of this premise for patterns of cooperation can be tested.

One can be either despairing or encouraged by this view of life. From the despondent position matters have to get bad before we make the most of our collaborative potential, while others will argue that it's just when life is troublesome that we are able to rise to the occasion by acting together. Putting aside such subjective responses the more objective conclusion is that cooperation seems to be scaled to adversity and responds adaptively to need.

In the very poorest environments, however, prosociality may break down altogether and a tentative conclusion from the arguments and evidence presented here is that quite small changes in adversity might have a large impact on cooperative sociality. Although this might work in either direction (Laughlin 1974, 1978) it requires theory on the dynamics of change to take this idea further. It is uncomfortable to accept, following

Turnbull for the Ik, that individualism in extreme adversity is adaptive; that *in extremis* selfishness is the favoured choice for survival. He may be right, but outside such extreme conditions the unusual human capacity for cooperation is at the heart of our sociality.

Acknowledgements

I am extremely grateful to John Baker whose comments resulted in many improvements and clarifications, including discussion of the comparability of environments when testing the ideas presented here. My thanks also to Tony Bennett, Matthew Johnson and Siobhán O'Sullivan for information and discussion, and to Quoc Vuong for guidance in preparing Figure 2.

Disclosure statement

The author states that there are no potential conflicts of interest.

Appendix: Evidence for the function shapes in **Figure 2**

This appendix provides evidence for the relationships between environmental quality and fitness or well-being proposed in Figure 2 in three parts: The right (diminishing returns) segment of the sigmoid curves; the left segment; and the convergence of the two curves (cooperation and non-cooperation) at both extremes. The evidence here is not selective but I have not attempted to find evidence for every kind of adversity.

The diminishing returns assumption is made in many behavioural ecological models, for example, for the way in which benefit to an offspring increases with parental investment (Trivers 1974); however, we are seeking empirical support here. Consider the common case of resource acquisition. In healthy animal populations in the wild (i.e. not *in extremis*), the rate of food intake generally increases with food density in a diminishing returns fashion (e.g. Goss-Custard et al. 2006) in accordance with foraging theory, as a consequence of the limiting effect of the time it takes to handle food (Stephens and Krebs 1986, 15). In addition, as an animal becomes satiated, further food intake brings increasingly less benefit no matter how much food the environment holds (as Winterhalder, Lu, and Tucker (1999, 304) also argue). This is why hungry and thirsty pigeons in the lab switch frequently between eating and drinking rather than, say, feeding to satiation before they start drinking (McFarland and Lloyd 1973); a unit reduction in thirst or hunger increases fitness more when the animal is further from its optimal internal state.

For humans, there is evidence of a diminishing marginal utility response of life expectancy to economic variables. This pattern is found across about 140 nations for the measure of national income per person (Wilkinson and Pickett 2010, Chapter 1; but see the main text discussion of Haushofer's 2013, per capita GDP measure) and within a single country, the US, for lifetime earnings (Cristia 2009). In the UK, life expectancy shows diminishing returns to various measures of deprivation, sometimes with a small concave upwards trend for those least deprived (Buck and Maguire 2015). Further, many studies show that subjective well-being, measured as life satisfaction or happiness, has a positive and diminishing marginal utility response to income, income change or wealth, for analyses both between countries, developed and developing (Frey and Stutzer 2002; Howell and Howell 2008), and within countries (Cummins 2000, a review of many studies; Graham and Pettinato 2001; Møller and Saris 2001, calculated from Tables I and II; Frey and Stutzer 2002). The causal relationship is from income to subjective well-being rather than in the opposite direction (Frey and Stutzer 2002; Gere and Schimmack 2017).

In a pioneering study of risk-sensitive foraging the feeding decisions of juncos are described by a sigmoid function. Experimental birds that would suffer a negative energy budget (*in extremis* conditions) if they fed at a predictable source are risk prone and show a concave upwards utility function, whereas those with a positive energy budget (i.e. a high environmental quality) are risk

Downloaded by [Dr John Lazarus] at 13:16 02 January 2018

averse and have a diminishing returns utility function (Caraco, Martindale, and Whittam 1980). These results have now been replicated many times in a variety of animal taxa, including two anthropological cases (reviewed by Winterhalder, Lu, and Tucker 1999).

A sigmoid function has also been found significantly to explain the relationship between an individual's quality and their resulting utility, outperforming linear and concave models, for primate sexual success as a function of rank and for social rank (which predicts reproductive success) as a function of hunting yield in Aché hunters (Kuznar 2002). The proximate mechanisms responsible for these relationships is unknown and, although the sigmoid function is offered by Kuznar as an expression of differential risk sensitivity, the mechanisms discussed earlier in this appendix may alternatively, or additionally, be responsible. In other anthropological studies risk-sensitive decision-making fits the predicted sigmoid pattern (Kuznar and Frederick 2003).

A sigmoid function is also suggested by the nature of abiotic factors influencing fitness (or well-being, for the rest of this paragraph), such as temperature, humidity and a great many factors for human populations. For such features (pollutants and suchlike aside), and for a particular species, there is an optimum value that maximizes an individual's fitness, with fitness declining above and below this optimal value: an inverted-U shape. Now, unless the transition through the optimal value is to make a sharp discontinuity, which is biologically implausible, it follows that the function approaches the optimal value, from both sides, in a diminishing returns fashion. Imagine now the environmental quality axis reconceptualized so that fitness reaches a maximum value at the extreme right, representing the optimal value of, say, temperature for the species. In this reconceptualization each point to the left of this optimal value represents a pair of temperatures – one below the optimum and another above it – that have an equal effect on fitness. The axis, therefore, represents adversity whether due to under- or over-shooting of the optimal environmental value and the redrawn function of temperature against adversity will be diminishing returns to the right. If the original inverted-U function is roughly normally distributed, and therefore bell-shaped, the adverse extremes, on the far left of the reconceptualized axis, will be concave upwards and the whole function will then be sigmoid. Son and Lewis (2005) provide a corroborating example relating temperature to survival for three life history stages of an insect. The functions are bell-shaped to the right (high temperature), where survival was extremely low or zero (*in extremis* conditions).

The data on severe food and water deprivation in rats (i.e. *in extremis* conditions, in contrast to natural feeding and drinking schedules (Siegel and Stuckey 1947)) show, as in the left segment of the sigmoid curve, a concave upwards relationship between environmental quality (the inverse of deprivation time) and fitness (the inverse of food or water intake after deprivation, a measure of distance from a homeostatic optimal state) (calculated from Clark 1958; Stellar and Hill 1952, for food and water, respectively). Andras and Lazarus (2005) assumed that the two curves might be convex upwards (diminishing returns) for the whole range of environmental quality but these data show this to be implausible.

Finally, I argue that the two sigmoid curves are unlikely to be parallel (in which case the benefit of cooperation would be a constant for all values of the environment) but are likely to converge at both extremes. For an individual in an extremely high-quality environment it seems likely that additional resources gained through cooperation would add little or nothing to fitness or well-being, either through sharing or by reciprocal altruistic exchanges. However, this may be too simplistic for the human case and I will consider it further in the text.

In the poorest environments, approaching *in extremis* conditions, there are several contexts in which convergence is likely. First, if cooperation consists of the acquisition and sharing of resources, at some point there are simply too few resources for cooperation – by physical help, skill and knowledge sharing, social influence or other means – to increase the resource sufficiently to compensate for the fact that it must be shared. Second, where cooperation consists of a series of altruistic exchanges, the strength of short-term need is sufficiently strong that failing to reciprocate becomes the favoured response. Third, in a life with many pressing needs the *opportunity* to cooperate may be compromised by time constraints (Siobhán O'Sullivan, personal communication, 8 January 2017).

Downloaded by [Dr John Lazarus] at 13:16 02 January 2018

References

Alexander, R. D. 1987. *The Biology of Moral Systems*. New York: Aldine de Gruyter.

Andras, P., J. Lazarus, and G. Roberts. 2007. "Environmental Adversity and Uncertainty Favour Cooperation." *BMC Evolutionary Biology* 7: 240. doi:10.1186/1471-2148-7-240.

Andras, P., and J. Lazarus. 2005. "Cooperation, Risk and the Evolution of Teamwork." In *Teamwork: Multi-Disciplinary Perspectives*, edited by N. Gold, 56–77, Basingstoke: Palgrave, Macmillan.

Ashworth, T. 1980. *Trench Warfare, 1914–1918: The Live and Let Live System*. New York: Holmes & Meier.

Axelrod, R. 1984. *The Evolution of Cooperation*. New York: Basic Books.

Axelrod, R., and W. D. Hamilton. 1981. "The Evolution of Cooperation." *Science* 211: 1390–1396. doi:10.1126/science.7466396.

Barclay, P. 2004. "Trustworthiness and Competitive Altruism can also Solve the "Tragedy of the Commons." *Evolution and Human Behavior* 25 (4): 209–220. doi:10.1016/j.evolhumbehav.2004.04.002.

Barkow, J. H., L. Cosmides, and J. Tooby, eds. 1992. *The Adapted Mind: Evolutionary Psychology and the Generation of Culture*. New York: Oxford University Press.

Barth, F. 1974. "On Responsibility and Humanity: Calling a Colleague to Account (With Reply by Turnbull)." *Current Anthropology* 15 (1): 99–103. doi:10.1086/201443.

Bateson, M., D. Nettle, and G. Roberts. 2006. "Cues of Being Watched Enhance Cooperation in a Real-World Setting." *Biology Letters* 2 (3): 412–414. doi:10.1098/rsbl.2006.05.09.

Bateson, P., and M. Mameli. 2007. "The Innate and the Acquired: Useful Clusters or a Residual Distinction from Folk Biology?." *Developmental Psychobiology* 49: 818–831. doi:10.1002/(ISSN)1098-2302.

Beidelman, T. O. 1973. "Review of '*The Mountain People*' by Colin M Turnbull." *Africa* 43: 170–171. doi:10.2307/1159341.

Binmore, K. 2005. *Natural Justice*. New York: Oxford University Press.

Binmore, K. 2007a. *Game Theory: A Very Short Introduction*. Oxford: Oxford University Press.

Binmore, K. 2007b. *Playing for Real: A Text on Game Theory*. New York: Oxford University Press.

Bowles, S., and H. Gintis. 2011. *A Cooperative Species: Human Reciprocity and Its Evolution*. Princeton: Princeton University Press.

Boyd, R., and P. J. Richerson. 1985. *Culture and the Evolutionary Process*. Chicago: University of Chicago Press.

Buck, D., and D. Maguire. 2015. *Inequalities in Life Expectancy: Changes over Time and Implications for Policy*. London: King's Fund.

Burch Jr., E. S. 2006. *Social Life in Northwest Alaska: The Structure of Iñupiaq Eskimo Nations*. Fairbanks: University of Alaska Press.

Byars, S. G., D. Ewbank, D. R. Govindaraju, and S. C. Stearns. 2010. "Natural Selection in a Contemporary Human Population." *Proceedings of the National Academy of Sciences* 107 (suppl 1): 1787–1792. doi:10.1073/pnas.0906199106.

Callaway, R. R. M., R. W. Brooker, P. Choler, Z. Kikvidze, C. J. Lortiek, R. Michalet, L. Paolini, et al. 2002. "Positive Interactions among Alpine Plants Increase with Stress". *Nature* 417: 844–847. 10.1038/nature00812.

Camerer, C. F. 2003. *Behavioral Game Theory*. Princeton: Princeton University Press.

Caraco, T., S. Martindale, and T. S. Whittam. 1980. "An Empirical Demonstration of Risk-Sensitive Foraging Preferences." *Animal Behaviour* 28: 820–830. doi:10.1016/S0003-3472(80)80142-4.

Casiro, J. 2006. "Argentine Rescuers: A Study on the "Banality of Good." *Journal of Genocide Research* 8 (4): 437–454. doi:10.1080/14623520601056281.

Chudek, M., and J. Henrich. 2011. "Culture-Gene Coevolution, Norm-Psychology and the Emergence of Human Prosociality." *Trends in Cognitive Sciences* 15: 218–226. doi:10.1016/j.tics.2011.03.003.

Clark, F. C. 1958. "The Effect of Deprivation and Frequency of Reinforcement on Variable-Interval Responding." *Journal of the Experimental Analysis of Behavior* 1 (3): 221–228. doi:10.1901/jeab.1958.1-221.

Downloaded by [Dr John Lazarus] at 13:16 02 January 2018

Colman, A. M. 1999. *Game Theory and Its Applications in the Social and Biological Sciences.* 2nd ed. London: Routledge.
Côté, S., J. House, and R. Willer. 2015. "High Economic Inequality Leads Higher-Income Individuals to Be Less Generous." *Proceedings of the National Academy of Sciences* 112 (52): 15838–15843. doi:10.1073/pnas.1511536112.
Craik, K. H. 2009. *Reputation: A Network Interpretation*. Oxford: Oxford University Press.
Cristia, J. P. 2009. "Rising Mortality and Life Expectancy Differentials by Lifetime Earnings in the United States." *Journal of Health Economics* 28 (5): 984–995. doi:10.1016/j.jhealeco.2009.06.003.
Cummins, R. A. 2000. "Personal Income and Subjective Well-Being: A Review." *Journal of Happiness Studies* 1 (2): 133–158. doi:10.1023/A:1010079728426.
Da Costa, M. K. B., C. D. de Melo, and P. F. M. Lopes. 2014. "Fisheries Productivity and its Effects on the Consumption of Animal Protein and Food Sharing of Fishers' and Non-Fishers' Families." *Ecology of Food and Nutrition* 53 (4): 453–470. doi:10.1080/03670244.2013.854781.
Davis, J. A. 1959. "A Formal Interpretation of the Theory of Relative Deprivation." *Sociometry* 22 (4): 280–296. doi:10.2307/2786046.
De Bono, M., D. M. Tobin, M. W. Davis, L. Avery, and C. I. Bargmann. 2002. "Social Feeding in *Caenorhabditis Elegans* is Induced by Neurons that Detect Aversive Stimuli." *Nature* 419: 899–903. doi:10.1038/nature01169.
Drenkard, E., and F. M. Ausubel. 2002. "*Psuedomonas* Biofilm Formation and Antibiotic Resistance are Linked to Phenotypic Variation." *Nature* 416: 740–743. doi:10.1038/416740a.
Dunbar, R. I. M. 2004. "Gossip in Evolutionary Perspective." *Review of General Psychology* 8 (2): 100–110. doi:10.1037/1089-2680.8.2.100.
Ellis, B. J., A. J. Figueredo, B. H. Brumbach, and G. L. Schlomer. 2009. "Fundamental Dimensions of Environmental Risk: The Impact of Harsh versus Unpredictable Environments on the Evolution and Development of Life History Strategies." *Human Nature* 20 (2): 204–268. doi:10.1007/s12110-009-9063-7.
Erev, I., G. Bornstein, and R. Galili. 1993. "Constructive Intergroup Competition as a Solution to the Free Rider Problem: A Field Experiment." *Journal of Experimental Social Psychology* 29 (6): 463–478. doi:10.1006/jesp.1993.1021.
Farr, J. A. 1975. "The Role of Predation in the Evolution of Social Behavior of Natural Populations of the Guppy, *Poecilia Reticulata (Pisces: Poeciliidea)*." *Evolution* 29: 151–158. doi:10.1111/j.1558-5646.1975.tb00822.x.
Fehr, E., and S. Gachter. 2002. "Altruistic Punishment in Humans." *Nature* 415 (6868): 137–140. doi:10.1038/415137a.
Frankenhuis, W. E., K. Panchanathan, and D. Nettle. 2016. "Cognition in Harsh and Unpredictable Environments." *Current Opinion in Psychology* 7: 76–80. doi:10.1016/j.copsyc.2015.08.011.
Frey, B. S., and A. Stutzer. 2002. "What Can Economists Learn from Happiness Research?." *Journal of Economic Literature* 40 (2): 402–435. doi:10.1257/jel.40.2.402.
Fritz, C. E., and H. B. Williams. 1957. "The Human Being in Disasters: A Research Perspective." *The Annals of the American Academy of Political and Social Science* 309 (1): 42–51. doi:10.1177/000271625730900107.
Gere, J., and U. Schimmack. 2017. "Benefits of Income: Associations with Life Satisfaction among Earners and Homemakers." *Personality and Individual Differences* 119: 92–95. doi:10.1016/j.paid.2017.07.004.
Gintis, H. 2003. "Solving the Puzzle of Prosociality." *Rationality and Society* 15 (2): 155–187. doi:10.1177/1043463103015002001.
Gintis, H., S. Bowles, R. Boyd, and E. Fehr. 2003. "Explaining Altruistic Behaviour in Humans." *Evolution and Human Behavior* 24 (3): 153–172. doi:10.1016/S1090-5138(02)00157-5.
Gintis, H., S. Bowles, R. Boyd, and E. Fehr, eds. 2005. *Moral Sentiments and Material Interests: The Foundations of Cooperation in Economic Life*. Cambridge, Massachusetts: MIT Press.
Goss-Custard, J. D., A. D. West, M. G. Yates, R. W. G. Caldow, R. A. Stillman, L. Bardsley, and J. Castilla. 2006. "Intake Rates and the Functional Response in Shorebirds (*Charadriiformes*) Eating Macro-Invertebrates." *Biological Reviews* 81: 501–529. doi:10.1017/S1464793106007093.

Downloaded by [Dr John Lazarus] at 13:16 02 January 2018

Graham, C., and S. Pettinato. 2001. “Happiness, Markets, and Democracy: Latin America in Comparative Perspective.” *Journal of Happiness Studies* 2 (3): 237–268. doi:10.1023/A:1011860027447.

Greenberg, E. P. 2003. “Tiny Teamwork.” *Nature* 424: 134. doi:10.1038/424134a.

Guala, F. 2012. “Reciprocity: Weak or Strong? What Punishment Experiments Do (And Do Not) Demonstrate.” *Behavioral and Brain Sciences* 35: 1–59. doi:10.1017/S0140525X11000069.

Gurven, M. 2004. “To Give and to Give Not: The Behavioral Ecology of Human Food Transfers.” *Behavioral and Brain Sciences* 27: 543–583. doi:10.1017/S0140525X04000123.

Haley, K. J., and D. M. T. Fessler. 2005. “Nobody’s Watching? Subtle Cues Affect Generosity in an Anonymous Economic Game.” *Evolution and Human Behavior* 26: 245–256. doi:10.1016/j.evolhumbehav.2005.01.002.

Hammerstein, P., ed. 2003. *Genetic and Cultural Evolution of Cooperation*. Cambridge, Massachusetts: MIT Press in cooperation with Dahlem University Press.

Haselton, M. G., and D. Nettle. 2006. “The Paranoid Optimist: An Integrative Evolutionary Model of Cognitive Biases.” *Personality and Social Psychology Review* 10 (1): 47–66. doi:10.1207/s15327957pspr1001_3.

Haushofer, J. 2013. “The Psychology of Poverty: Evidence from 43 Countries.” Retrieved on 18 September 2017 from http://www.princeton.edu/~joha/publications/Haushofer_2013.pdf.

Heine, B. 1985. “The Mountain People: Some Notes on the Ik of North-Eastern Uganda.” *Africa* 55 (1): 3–16. doi:10.2307/1159836.

Henrich, J., R. Boyd, S. Bowles, C. Camerer, E. Fehr, and H. Gintis, eds. 2004. *Foundations of Human Sociality: Economic Experiments and Ethnographic Evidence from Fifteen Small-Scale Societies*. Oxford: Oxford University Press.

Hill, R. A., and P. C. Lee. 1998. “Predation Risk as an Influence on Group Size in Cercopithecoid Primates: Implications for Social Structure.” *Journal of Zoology* 245: 447–456. doi:10.1111/j.1469-7998.1998.tb00119.x.

Howell, R. T., and C. J. Howell. 2008. “The Relation of Economic Status to Subjective Well-Being in Developing Countries: A Meta-Analysis.” *Psychological Bulletin* 134 (4): 536–560. doi:10.1037/0033-2909.134.4.536.

Hugh-Jones, D., and J. Ooi. 2017. *Where Do Fairness Preferences Come From? Norm Transmission in a Teen Friendship Network*. University of East Anglia. 2 October 2017. Downloaded from https://sites.google.com/site/davidhughjones.

Kaplan, H., K. Hill, and A. M. Hurtado. 1990. “Risk, Foraging and Food Sharing among the Ache.” In *Risk and Uncertainty in Tribal and Peasant Economies*, edited by E. Cashdan, 107–143. San Francisco: Westview Press.

Kelly, R. L. 2013. *The Lifeways of Hunter-Gatherers: The Foraging Spectrum*. 2nd ed. Cambridge: Cambridge University Press.

Knight, J. A. 1976. “On the Ik and Anthropology - Further Note.” *Current Anthropology* 17 (4): 777. doi:10.1086/201839.

Korndörfer, M., B. Egloff, and S. C. Schmukle. 2015. “A Large Scale Test of the Effect of Social Class on Prosocial Behavior.” *PloS ONE* 10 (7): e0133193. doi:10.1371/journal.pone.0133193.

Krause, J., and G. D. Ruxton. 2002. *Living in Groups*. Oxford: Oxford University Press.

Krupka, E. L., and R. A. Weber. 2013. “Identifying Social Norms Using Coordination Games: Why Does Dictator Game Sharing Vary?.” *Journal of the European Economic Association* 11 (3): 495–524. doi:10.1111/jeea.12006.

Kurzban, R. 2001. “The Social Psychophysics of Cooperation: Nonverbal Communication in a Public Goods Game.” *Journal of Nonverbal Behavior* 25 (4): 241–259. doi:10.1023/A:1012563421824.

Kuznar, L. A. 2002. “Evolutionary Applications of Risk Sensitivity Models to Socially Stratified Species: Comparison of Sigmoid, Concave, and Linear Functions.” *Evolution and Human Behavior* 23 (4): 265–280. doi:10.1016/S1090-5138(01)00105-2.

Kuznar, L. A., and W. G. Frederick. 2003. “Environmental Constraints and Sigmoid Utility: Implications for Value, Risk Sensitivity, and Social Status.” *Ecological Economics* 46 (2): 293–306. doi:10.1016/S0921-8009(03)00167-8.

Downloaded by [Dr John Lazarus] at 13:16 02 January 2018

Laland, K. N., J. Odling-Smee, and S. Myles. 2010. "How Culture Shaped the Human Genome: Bringing Genetics and the Human Sciences Together." *Nature Reviews. Genetics* 11 (2): 137–149. doi:10.1038/nrg2734.

Laughlin Jr., C. D. 1974. "Deprivation and Reciprocity." *Man (N.S.)* 9 (3): 380–396. doi:10.2307/2800691.

Laughlin Jr., C. D., and I. A. Brady. 1978. "Introduction: Diaphasis and Change in Human Populations." In *Extinction and Survival in Human Populations*, edited by C. D. Laughlin Jr. and I. A. Brady, 1–48. New York: Columbia University Press.

Laughlin, Jr., C. 1978. "Adaptation and Exchange in So: A Diachronic Study of Deprivation." In *Extinction and Survival in Human Populations*, edited by C. D. Laughlin Jr. and I. A. Brady, 76–94. New York: Columbia University Press.

Lazarus, J. 2003. "Let's Cooperate to Understand Cooperation." *Behavioral and Brain Sciences* 26 (2): 169–170. doi:10.1017/S0140525X03390053.

Lieberman, D., J. Tooby, and L. Cosmides. 2003. "Does Morality Have a Biological Basis? An Empirical Test of the Factors Governing Moral Sentiments Relating to Incest." *Proceedings of the Royal Society B* 270: 819–826. doi:10.1098/rspb.2002.2290.

Lim, D., and D. DeSteno. 2016. "Suffering and Compassion: The Links among Adverse Life Experiences, Empathy, Compassion, and Prosocial Behavior." *Emotion* 16 (2): 175–182. doi:10.1037/emo0000144.

Lim, D. 2017. "The Influence of Suffering, Social Class, and Social Power on Prosociality: An Empirical Review." In *Empathy: An Evidence-Based Interdisciplinary Perspective*, edited by M. Kondo, 81–92. London: Intech.

Low, B. S. 1990. "Human Responses to Environmental Extremeness and Uncertainty: A Cross-Cultural Perspective." In *Risk and Uncertainty in Tribal and Peasant Economies*, edited by E. Cashdan, 229–255. San Francisco: Westview Press.

Mameli, M., and P. Bateson. 2006. "Innateness and the Sciences." *Biology and Philosophy* 21: 155–188. doi:10.1007/s10539-005-5144-0.

Maynard Smith, J. 1982. *Evolution and the Theory of Games*. Cambridge: Cambridge University Press.

McFarland, D. J., and I. H. Lloyd. 1973. "Time-Shared Feeding and Drinking." *The Quarterly Journal of Experimental Psychology* 25 (1): 48–61. doi:10.1080/14640747308400322.

Milinski, M., D. Semmann, T. C. M. Bakker, and H.-J. Krambeck. 2001. "Cooperation through Indirect Reciprocity: Image Scoring or Standing Strategy?" *Proceedings of the Royal Society* 268: 2495–2501. doi:10.1098/rspb.2001.1809.

Møller, V., and W. E. Saris. 2001. "The Relationship between Subjective Well-Being and Domain Satisfactions in South Africa." *Social Indicators Research* 55 (1): 97–114. doi:10.1023/A:1010851412273.

Nettle, D. 2009. "Beyond Nature versus Culture: Cultural Variation as an Evolved Characteristic." *Journal of the Royal Anthropological Institute (N.S.)* 15: 223–240. doi:10.1111/jrai.2009.15.issue-2.

Nettle, D. 2010. "Why are There Social Gradients in Preventative Health Behavior? A Perspective from Behavioral Ecology." *PloS ONE* 5 (10): e13371. doi:10.1371/journal.pone.0013371.

Nettle, D. 2015. *Tyneside Neighbourhoods: Deprivation, Social Life and Social Behaviour in one British City*. Cambridge, UK: Open Book Publishers.

Nettle, D. 2017. "Does Hunger Contribute to Socioeconomic Gradients in Behavior?." *Frontiers in Psychology* 8: 358. doi:10.3389/fpsyg.2017.00358.

Nettle, D., A. Colléony, and M. Cockerill. 2011. "Variation in Cooperative Behaviour within a Single City." *PloS ONE* 6 (10): e26922. doi:10.1371/journal.pone.0026922.

Nettle, D., G. V. Pepper, R. Jobling, and K. B. Schroeder. 2014. "Being There: A Brief Visit to a Neighbourhood Induces the Social Attitudes of that Neighbourhood." *Peer Journal* 2: e236. doi:10.7717/peerj.236.

Nowak, M. A., and K. Sigmund. 1993. "A Strategy of Win-Stay, Lose-Shift that Outperforms Tit-For-Tat in the Prisoner's Dilemma Game." *Nature* 364: 56–58. doi:10.1038/364056a0.

Ostrom, E. 1990. *Governing the Commons: The Evolution of Institutions for Collective Action*. Cambridge: Cambridge University Press.

Downloaded by [Dr John Lazarus] at 13:16 02 January 2018

Pepper, G. V., and D. Nettle. 2017. "The Behavioural Constellation of Deprivation: Causes and Consequences." *Behavioral and Brain Sciences* 1–72. doi:10.1017/S0140525X1600234X.

Piff, P. K. 2014. "Wealth and the Inflated Self: Class, Entitlement, and Narcissism." *Personality and Social Psychology Bulletin* 40 (1): 34–43. doi:10.1177/0146167213501699.

Piff, P. K., M. W. Kraus, S. Côté, B. H. Cheng, and D. Keltner. 2010. "Having Less, Giving More: The Influence of Social Class on Prosocial Behavior." *Journal of Personality and Social Psychology* 99 (5): 771. doi:10.1037/a0020092.

Pinker, S. 2002. *The Blank Slate: The Modern Denial of Human Nature*. London: Allen Lane The Penguin Press.

Puurtinen, M., S. Heap, and T. Mappes. 2015. "The Joint Emergence of Group Competition and Within-Group Cooperation." *Evolution and Human Behavior* 36 (3): 211–217. doi:10.1016/j.evolhumbehav.2014.11.005.

Puurtinen, M., and T. Mappes. 2009. "Between-Group Competition and Human Cooperation." *Proceedings of the Royal Society of London B: Biological Sciences* 276 (1655): 355–360. doi:10.1098/rspb.2008.1060.

Richerson, P. J., R. Boyd, and J. Henrich. 2010. "Gene-Culture Coevolution in the Age of Genomics." *Proceedings of the National Academy of Sciences* 107 (Supplement 2): 8985–8992. doi:10.1073/pnas.0914631107.

Richerson, P. J., and R. Boyd. 2005. *Not by Genes Alone: How Culture Transformed Human Evolution*. Chicago: University of Chicago Press.

Ridley, M. 1997. *The Origins of Virtue*. London: Penguin.

Roberts, G. 1998. "Competitive Altruism: From Reciprocity to the Handicap Principle." *Proceedings of the Royal Society of London B* 265:427–431.

Roberts, G. 2005. "Cooperation through Interdependence." *Animal Behaviour* 70: 901–908. doi:10.1016/j.anbehav.2005.02.006.

Schroeder, K. B., G. V. Pepper, and D. Nettle. 2014. "Local Norms of Cheating and the Cultural Evolution of Crime and Punishment: A Study of Two Urban Neighborhoods." *Peer Journal* 2: e450. doi:10.7717/peerj.450.

Seghers, B. H. 1974. "Schooling Behaviour in the Guppy (*Poecilia reticulata*): An Evolutionary Response to Predation." *Evolution* 28 (3): 486–489.

Siegel, P. S., and H. L. Stuckey. 1947. "The Diurnal Course of Water and Food Intake in the Normal Mature Rat." *Journal of Comparative and Physiological Psychology* 40 (5): 365–370. doi:10.1037/h0062185.

Smaldino, P. E., J. C. Schank, and R. McElreath. 2013. "Increased Costs of Cooperation Help Cooperators in the Long Run." *American Naturalist* 181 (4): 451–463. doi:10.1086/669615.

Son, Y., and E. E. Lewis. 2005. "Modelling Temperature-Dependent Development and Survival of *Otiorhynchus sulcatus* (Coleoptera: Curculionidae)." *Agricultural and Forest Entomology* 7 (3): 201–209. doi:10.1111/j.1461-9555.2005.00260.x.

Spinks, A. C., J. U. M. Jarvis, and N. C. Bennett. 2000. "Comparative Patterns of Philopatry and Dispersal in Two Common Mole-Rat Populations: Implications for the Evolution of Mole-Rat Sociality." *Journal of Animal Ecology* 69: 224–234. doi:10.1046/j.1365-2656.2000.00388.x.

Standing, G. 2011. *The Precariat: The New Dangerous Class*. London: Bloomsbury.

Stein, A. A. 1976. "Conflict and Cohesion: A Review of the Literature." *Journal of Conflict Resolution* 20 (1): 143–172. doi:10.1177/002200277602000106.

Stellar, E., and J. H. Hill. 1952. "The Rat's Rate of Drinking as a Function of Water Deprivation." *Journal of Comparative and Physiological Psychology* 45 (1): 96–102. doi:10.1037/h0062150.

Stephens, D. W., and J. R. Krebs. 1986. *Foraging Theory*. Princeton, New Jersey: Princeton University Press.

Tec, N. 1986. *When Light Pierced the Darkness: Christian Rescue of Jews in Nazi-Occupied Poland*. New York: Oxford University Press.

Tomasello, M. 2009. *Why We Cooperate. Based on the 2008 Tanner Lectures on Human Values at Stanford*. Cambridge, Massachusetts: A Boston Review Book. The MIT Press.

Downloaded by [Dr John Lazarus] at 13:16 02 January 2018

Tooby, J., and L. Cosmides. 1990. "The Past Explains the Present: Emotional Adaptations and the Structure of Ancestral Environments." *Ethology and Sociobiology* 11 (4–5): 375–424. doi:10.1016/0162-3095(90)90017-Z.
Trivers, R. L. 1974. "Parent-Offspring Conflict." *American Zoologist* 14: 249–264. doi:10.1093/icb/14.1.249.
Turnbull, C. M. [1972] 1974. *The Mountain People*. London: Pan Books (Picador).
Turnbull, C. M. 1966. *Tradition and Change in African Tribal Life*. Cleveland: World.
Turnbull, C. M. 1967. "The Ik: Alias the Teuso." *Uganda Journal* 31 (1): 63–71.
Turnbull, C. M. 1975. "Reply." *Current Anthropology* 16 (3): 354–358.
Turnbull, C. M. 1976. "Turnbull Replies." *Rain* 16 (Oct): 4–6. doi:10.2307/3031968.
Turnbull, C. M. 1978. "Rethinking the Ik: A Functional Non-Social System." In *Extinction and Survival in Human Populations*, edited by C. D. Laughlin Jr and I. A. Brady, 49–75. New York: Columbia University Press.
Westermarck, E. A. 1921. *The History of Human Marriage*. 5th ed. London: Macmillan.
Wilkinson, R., and K. Pickett. 2010. *The Spirit Level: Why Equality is Better for Everyone*, 2009. London: Penguin.
Wilson, P. J., G. McCall, W. R. Geddes, A. K. Mark, J. E. Pfeiffer, J. B. Boskey, and C. M. Turnbull. 1975. "More Thoughts on the Ik and Anthropology [And Reply]." *Current Anthropology* 16 (3): 343–358. doi:10.1086/201566.
Winterhalder, B. 1986. "Diet Choice, Risk, and Food Sharing in a Stochastic Environment." *Journal of Anthropological Archaeology* 5 (4): 369–392. doi:10.1016/0278-4165(86)90017-6.
Winterhalder, B., F. Lu, and B. Tucker. 1999. "Risk-Sensitive Adaptive Tactics: Models and Evidence from Subsistence Studies in Biology and Anthropology." *Journal of Archaeological Research* 7 (4): 301–348. doi:10.1007/BF02446047.

Downloaded by [Dr John Lazarus] at 13:16 02 January 2018
