# Supplementary material for: Environmental adversity is associated with lower investment in collective actions
Source: PLoS One. 2020 Jul 30;15(7):e0236715. doi: 10.1371/journal.pone.0236715 (PMC7392252; doi:10.1371/journal.pone.0236715)
Supplement: Attachment — Submitted filename: Response to Reviewers.docx [file pone.0236715.s016.docx]

**Subject:** Resubmitted Manuscript PONE-D-20-00738 in Draft

Dear Dr. Capraro,

Thank you for your helpful review of our manuscript and for providing us with the opportunity to revise and resubmit our work. We wish to thank the two reviewers for their positive evaluation of the work, their helpful comments and advice. Below we provide a point-by-point reply to the reviewers’ comments, with changes to the paper placed in italics. We addressed all the reviewers’ comments and made significant changes to the introduction, clarified several topics and provided additional supportive data.

We hope you will find this version of the paper acceptable.

With kind regards,

Niels Lettinga, Pierre O. Jacquet, Jean-Baptiste André, Nicolas Baumard, Coralie Chevallier

***Comments to the Authors***

*1. Is the manuscript technically sound, and do the data support the conclusions?*

*The manuscript must describe a technically sound piece of scientific research with data that supports the conclusions. Experiments must have been conducted rigorously, with appropriate controls, replication, and sample sizes. The conclusions must be drawn appropriately based on the data presented.

Reviewer #1: Yes
Reviewer #2: Yes*

*2. Has the statistical analysis been performed appropriately and rigorously?

Reviewer #1: I Don't Know
Reviewer #2: Yes*

*3. Have the authors made all data underlying the findings in their manuscript fully available?*

*The* [*PLOS Data policy*](http://www.plosone.org/static/policies.action#sharing) *requires authors to make all data underlying the findings described in their manuscript fully available without restriction, with rare exception (please refer to the Data Availability Statement in the manuscript PDF file). The data should be provided as part of the manuscript or its supporting information, or deposited to a public repository. For example, in addition to summary statistics, the data points behind means, medians and variance measures should be available. If there are restrictions on publicly sharing data—e.g. participant privacy or use of data from a third party—those must be specified.

Reviewer #1: Yes
Reviewer #2: Yes*

*4. Is the manuscript presented in an intelligible fashion and written in standard English?*

*PLOS ONE does not copyedit accepted manuscripts, so the language in submitted articles must be clear, correct, and unambiguous. Any typographical or grammatical errors should be corrected at revision, so please note any specific errors here.

Reviewer #1: Yes
Reviewer #2: Yes*

**REVIEWER #1**

*Reviewer #1: Environmental harshness is associated with lower investment in collective actions

Lettinga, N., Jacquet, P.O., André, J-B., Baumard, N., Chevallier, C.

Reviewer: John Lazarus

Limitations of review. I am not expert in structural equation modelling and I do not have experience working with either of the data sets used here.*

***General comments*** *1. This is a valuable study in a currently lively and somewhat controversial area of research. It is particularly useful in measuring associations of prosociality with both environmental harshness and measures predicted by life history strategy (LHS) theory.

2. The paper tests the hypotheses that prosociality is reduced in adults both in a harsher current environment and when raised in a harsher environment during childhood. The paper is clearly written, the Introduction covers the relevant literature well and the Discussion section is commendable for the way it considers limitations of the study; problems of causal inference from correlational data; and alternative evolutionary explanations for the findings.

3. Throughout the paper the authors describe various behavioural measures examined by others, and by themselves in this paper, as ‘cooperation’. In evolutionary biology cooperation is defined as a social action resulting in a benefit to both (all) parties in the interaction. The behaviours analysed here are volunteering and political action, behaviours that would be defined in evolutionary biology as ‘altruism’; that is, actions resulting in a benefit to the recipient(s) and a cost to the self, and the authors agree that these two behaviours do have this cost-benefit structure (lines 187, 189-90 and 475 [later numerical references are also to line numbers]). Similarly some behavioural measures in previous studies cited here as cooperative (e.g. reference 30) are actually altruistic (e.g. volunteering and charitable giving).*

*Since this paper is testing hypotheses within the discipline of evolutionary human studies it is important that the terminology is correct. This is far from being a dry terminological matter; rather it is central to the hypotheses tested here, as I shall go on to argue. The authors are testing the idea that in harsh environments there is a ‘need to favour immediate over long-term benefits’ (54) and they state that ‘cooperation is a future-oriented strategy: in the short term, it is more advantageous to reap immediate, smaller, but more certain benefits by cooperating less’ (62-64).*

*I have a number of comments related to this point:*

- *(a) The behavioural measures employed here – volunteering and political action – are altruistic not cooperative as defined above. Consequently they are suitable for testing the hypothesis that they will be less evident in harsh environments, but this is because they are immediately costly, and not, as argued in the paper, because benefits from them are delayed.*
- *(b) I do not agree with the claim quoted above (62-64, also 19-20) that cooperation is necessarily a ‘future-oriented strategy’. It is future oriented in the case of reciprocal altruism where, after an exchange of altruistic acts between two individuals, both have gained a net benefit (but is only future-oriented if the focal individual is the first altruist). (Volunteering and political action, in themselves, do not fall into this category – rather they are altruistic – although one can imagine scenarios in which they are followed by a reciprocal benefit.) But where the cooperation involves joint simultaneous activity there is an immediate benefit to both parties (compared to not cooperating).*
- *(c) In consequence of this last point, and following the arguments of the authors, simultaneous cooperation, as defined here, would be predicted to be more common under harsher conditions since it provides an immediate benefit in comparison with the decision to refrain from cooperation.*
- *(d) In using a ‘proxy’ measure for cooperation (e.g. 95-6) it is necessary that the proxy satisfies the definition of cooperation given above. I do not mean here the distinction between behavioural and attitudinal measures (472-3), which is, of course, important. Rather, I mean that whether it is a behavioural or an attitudinal measure it should satisfy the above definition of cooperation (which for an attitude would refer to an imagined cost-benefit structure).*
- *(e) I suggest that the authors look again at their use of the terms ‘cooperation’ and ‘altruism’, and their derivatives, in light of these comments. At the same time it would be useful also to check use of the terms ‘prosociality’ and ‘collective action’. ‘Prosociality’ refers to a social action providing a benefit to the recipient and therefore includes both altruism and cooperation, as defined above. I would like to see a definition in the paper of ‘collective action’ since it is used a number of times. My next point argues, more generally, for the importance of clear definitions and distinctions between the different types of prosocial behavior for this field of research.*

We thank the reviewer for highlighting this issue. We believe that the confusion arises from the different definitions used. The reviewer states that “in evolutionary biology cooperation is defined as a social action resulting in a benefit to both (all) parties in the interaction”. This definition is also given in Lazarus [1] but without a reference, therefore we were not able to go back to the original source. Our definition of cooperation (and related terms) is based on West et al. [2]’s work, which is standard in evolutionary biology. Cooperation is defined by West et al. [2,3] as “a behavior that provides a benefit to another individual (recipient), and the evolution of which has been dependent on its beneficial effect for the recipient”. In this definition, nothing is said about the costs and benefits to the actor. Cooperation has two subcategories: altruistic and mutually beneficial behaviors [2]. Altruism is defined as “a behavior that is costly to the actor and beneficial to the recipient or recipients. Costs and benefits are defined on the basis of the lifetime direct fitness consequences of a behavior” [2,4]. Mutual benefit is defined by West et al. [2,3] as “a behavior that is beneficial to both the actor and the recipient”. Thus, cooperation as defined by the reviewer is less inclusive (i.e., only mutually beneficial behaviors) than as defined by West et al. [2] (i.e., mutually beneficial and altruistic behaviors). We are particularly interested in mutually beneficial cooperative behaviors, where the actor incurs immediate costs and the benefits are delayed (e.g., via social reputation), which was not stated clearly in our manuscript.

It is true that we stated that we looked for behaviors in the European Values Study that involve a concrete cost for the agent and a benefit for others, which would thus be defined as altruism according to the definitions provided by West et al. [2]. Volunteering and political actions indeed involve immediate costs to the agent and benefits for others. However, the actor also receives delayed benefits (e.g., via increased social reputation). From this perspective, volunteering and political actions would be described as mutually beneficial behaviors. Volunteering, for example, is positively associated with concern for social reputation or image [5] and is a means of obtaining human capital that will yield returns in the labor market [6,7]. Political action, for example voting, has also been shown to correlate with higher social reputation [8,9]. Thus, volunteering and political actions are cooperative activities (i.e., mutually beneficial).

We fully agree with the reviewer that when “cooperation involves joint simultaneous activity there is an immediate benefit to both parties”, it is not necessarily a future-oriented strategy because the benefits are immediate for both parties. We also agree with the reviewer that if an action is simultaneously beneficial to both (all) parties it would be beneficial to perform that action (except for possible opportunity costs), irrespective of under what conditions (adverse or otherwise) that action is made. However, because the cooperative behaviors we studied meet West et al’s definition, in the sense that they are behaviors “that provide a benefit to another individual (recipient)” [2,3], that are immediately costly for the actor, and that provide benefits in the long-run (e.g., via social reputation), then the form of cooperation we are interested in can be seen as a future-oriented strategy. As other future-oriented behaviors, the characteristics of the environment (e.g., adversity) may affect the degree to which it is beneficial to cooperate or not. Because people in adverse environments appear to favor immediate over long-term benefits [10], it makes sense that they would cooperate less (following the logic that is provided in the manuscript).

The point regarding using collective action as a proxy for cooperation is a very important point indeed, and one that we failed to address in our manuscript. We thank the reviewer for highlighting this issue. Collective action is defined as situations in which “multiple individuals pay the cost of contributing to a group project and share in the resulting benefit” [11,12]. In both cooperation and collective actions, the actors immediately pay a cost and receive a possible delayed benefit. Thus, collective action is the n-person equivalent of cooperation. Therefore, we think that collective action is a good proxy for cooperation.

In order to clarify the above misunderstanding, we include a definition of cooperation and collective action in the introduction of the revised manuscript on p.5 and p.6, respectively and we include the information about volunteering and political action as mutually beneficial behaviors on p.10 and on p.11 of the revised manuscript.

*Cooperation is defined as “a behavior that provides a benefit to another individual (recipient), and the evolution of which has been dependent on its beneficial effect for the recipient” [61,62]. Cooperation has two subcategories: altruistic and mutually beneficial behaviors [62]. We are particularly interested in mutually beneficial cooperative behaviors, where the actor incurs immediate costs and the benefits are delayed. In this framework, cooperation is a future-oriented strategy [63,64].*

*Collective action can indeed be defined as “multiple individuals pay the cost of contributing to a group project and share in the resulting benefit” [84,85].*

*We scanned the European Values Study to identify variables that had previously been found to successfully predict real-life cooperation [33] and that tapped actual behaviors involving concrete costs and delayed benefits for the agent and benefits for others. Two sets of variables met our a priori defined criteria: how much someone volunteers (scale 0-15) and how often someone participates in political actions that might benefit the community, without receiving financial compensation (scale 0-5) (items are detailed in S2 Table of the Supporting Information). People who volunteer or take part in political actions indeed incur concrete costs, including time and energy, to engage in actions that benefit others. Furthermore, volunteering and political actions also have delayed benefits for the actor. Volunteering, for example, is positively associated with concern for social reputation or image [92] and is a means of obtaining human capital that will yield returns in the labor market [93,94]. Political action, for example voting, has also been shown to correlate with higher social reputation [95,96].*

*4. Following on from comment (e), it seems to me that the discrepant findings in the literature about the effect of environmental harshness on prosociality (the authors refer to this at 42-6) may be partly a function of the literature not distinguishing carefully enough between different types of social interaction, with potentially different cost-benefit structures for the players; broadly cooperation versus altruism.

I declare an interest here. I have a paper (Lazarus 2017, and included with this review), one not cited by the authors, in which I review evidence from humans and other taxa that cooperation (defined, as above, in evolutionary biology) may be an inverted-U function of environmental adversity (i.e. harshness). The review is accompanied by an explanatory evolutionary model that differs from the ideas discussed in the present paper (i.e. selection for present-focused decision making in harsh conditions). The model is intended to supplement, and not to replace, explanations based on present-future trade-offs. The authors may wish to refer to my paper.

My paper also discusses the point made in the paragraph before last and I quote the passage here (Lazarus 2017, section 6, p. 590):

Other forms of prosociality*

*Cooperative behaviour shares motivational and emotional features, and evolutionary and cultural origins, with related prosocial behaviours, beliefs and attitudes, and the influence of adversity has been studied here too. At the level of individual differences those with more adverse life experience exhibit more empathy, compassion and generosity in charitable giving and aid to a stranger (Lim and DeSteno 2016; Lim 2017). The influence of social class on prosociality is controversial, with recent conflicting findings for a range of measures: utilitarianism, empathy, feelings of entitlement, narcissism, theft, lying, cheating, helpfulness, generosity, trust, trustworthiness, volunteering and charitable donation. Some studies find greater prosociality in higher classes (Korndörfer, Egloff, and Schmukle 2015) but others find the opposite class effect (Côté, House, and Willer 2015), to mention just two of the most recent reports. In understanding these apparently conflicting findings it will be helpful to develop more tailored predictions for each measure, as attempted here for cooperation. If the inverted-U relationship proposed here between environmental quality and cooperation should hold also for some of these other forms of prosociality this might resolve some of the (apparent) inconsistencies in the data, since conflicting studies could be situated on opposite sides of the environmental inflection point. As others have noted it is also important to take account of the methodology by which these results have been obtained, from behavioural observations and experiments to self-report, in both the lab and the real world, since they each have their own psychological influences.

I invite the authors to engage with these ideas.*

This is a very interesting suggestion indeed. We are unsure whether the inconsistent findings in the literature are due to not distinguishing enough between different types of social interaction. This is an interesting avenue for further research; however it is not the focus of our study. Such a research question would require additional data that we do not possess.

Elsewhere, we have argued that the inconsistent findings in the literature could be due to the way cooperation is measured, which is also mentioned in Lazarus [1]: “As others have noted it is also important to take account of the methodology by which these results have been obtained, from behavioural observations and experiments to self-report, in both the lab and the real world, since they each have their own psychological influences”. On the one hand, when cooperation is measured via self-reported questionnaires or in field experiments, the pattern of results is rather consistent and suggestive of an association between adverse environments and decreased cooperation. The reviewer mentions Côté et al. [13] as an example for the relationship between adverse environments and increased cooperation. However, Schmukle et al. [14] failed to replicate the findings of Côté et al. [13] using three large cross-cultural and representative samples (average N over 3 studies = 20.011). On the other hand, when cooperation is measured using behavioral experiments (i.e., economic games and actual behavior in various settings under scrutiny of the experimenter), the results are much more mixed. Because Lim and DeSteno [15] also use behavioral experiments that might be the reason why they find a relationship between severe adversity and more cooperation, which adds to the mixed findings in the literature.

Because we measure cooperation via self-reported questionnaires, we expected that we would find an association between adverse environments and decreased cooperation, which we in fact did. We have added the arguments for the inconsistent findings in the introduction of the revised manuscript on p.3 and p.4. Furthermore, we mention the proposed inverted U relationship by Lazarus [1] in the discussion on p.27.

*However, whether cooperation is decreased or increased in adverse environments is still debated. In theory, the relationship between environmental adversity and cooperation could go either way [30]. On the one hand, adverse environments might correlate with increased cooperation as a means to mitigate direct survival threats, such as pooling material resources to reduce the impact of external events like famine [31,32]. On the other hand, adverse environments might correlate with decreased cooperation because adversity constrains people to focus on trying to meet their individual needs [30].*

*The existing empirical evidence is currently inconclusive. When cooperation is measured via self-reported questionnaires or in field experiments, studies usually report an association between adverse environments and decreased cooperation. Notably, Korndörfer et al. [33] and Schmukle et al. [34] found a negative correlation between lower social class and cooperative behaviors (e.g., donating, volunteering, helping) using questionnaire data from large and representative international samples (average N over 9 studies = 20.243). Using field experiments, Andreoni et al. [35] and Nettle et al. [36] also found that lower social class correlates with decreased cooperation.*

*When cooperation is measured using behavioral experiments (i.e., economic games and actual behavior in various settings under scrutiny of the experimenter), the results are much more mixed. Some studies report an association between adverse environments and decreased cooperation [33,34,36–38], but other studies find the opposite pattern [39–41] or no effect at all [42,43].*

*The reason why these behavioral experiments give mixed results is unclear, but several explanations have been put forward. First, some of these studies have small sample sizes, which may explain, for example, why Piff et al. [41], who found a positive relation between environmental adversity and cooperation, was not replicated in a high-powered pre-registered study [42]. Second, these studies usually only use one economic game, which may lower their generalizability [44]. In accordance with this idea, McAuliffe et al. [45] found that single economic games do not correlate with real-life cooperation, but a general factor based on several economic games does. Finally, there is increasing evidence that economic games lack ecological validity [46].*

***Detailed comments*** *Title: Since the LHS variables play a major role in the association between environmental harshness and the prosociality variables I suggest that LHS is added to the title.*

We thank the reviewer for this suggestion. We have a more narrow focus on the reproduction-maintenance trade-off specifically, not life-history theory in general. Therefore we will keep the title without a reference to life-history theory.

*76. ‘By contrast’ with the previous sentence these references are expected to deal with the effect of the experience of safe conditions during human childhood. However, two seem not to be relevant: [54] is a comparative mammalian paper and [60] is a study of guppies.*

We have removed those two references.

*121 and 338. Provide references or web links to the two data sets.*

We have added the two references.

*123. The countries in the survey: it would be useful, in the supplementary material, to provide lists of the countries in each study uniquely, and in both studies. Country differences may help to explain some differences in the results of the two studies, and will also be useful for comparison with future studies.*

We have included a table with descriptive statistics for each country in the Supporting Information (S1 Table for the EVS and S5 Table for the WVS).

*134. ‘in’ should read ‘into’.*

We have changed this.

*135-7 & 370-72. ‘In both subsamples, we excluded participants with too many empty cells (more than two standard deviations from their respective subsample mean)’. I find this unclear; ‘empty cells’ seems to refer to missing data whereas the passage in parentheses seems to refer to outliers.*

Empty cells indeed refer to missing data. Respondents with too much missing data (the criterion was set to more than two standard deviations from a respondent’s respective subsample mean) were excluded. We have rephrased this to make it clearer.

*151. ‘parent’s’ should read ‘parents’. ‘end’ should read ‘ends’, both here and in the figures.*

We have changed this.

*166. This should probably read ‘health costs are induced’.*

We have changed this.

*177. ‘no’ should read ‘not’.*

We have changed this.

*248 and 403. Discovery and replication samples in the two studies. In the Results section of each study there is analysis of just one set of data. It is not stated whether this is the discovery sample, the replication sample, or the two combined. If it is one of the two samples then the second should be added, or comparison made with the first. If it is the two combined then any differences between the two samples should be described.*

The results that are presented in the main manuscript are from the replication sample, this is now stated more clearly on p.13 for the EVS and on p.21 for the WVS. The results of the discovery sample are now included in the Supporting Information (S1 Text for the EVS and S4 Text for the WVS).

*255-9. Additional analysis with imputed data, to deal with the problem of missing responses. ‘The results of this analysis are consistent with the results obtained using listwise deletions (see S1 Text in the Supporting Information)’ (257-9). In contrast to this statement in the paper there is an important difference in the results of the two analyses. In the main text analysis the authors conclude from a significant negative association between childhood harshness and collective action (309 and Figure 2) that ‘This implies that a harsher childhood environment is associated with less investment in collective actions later in life’ (309-10). However, the same conclusion is drawn from the additional analysis although now the same correlation is significant and positive (S1 Text, p. 3, lines 3-4 and Figure S1). This requires Discussion in the main text, particularly since it represents a core prediction of the paper.*

The reviewer is right to point this out; we indeed made a mistake here. We have changed the results section on p.13 and on p.14 and the Supporting Information (see S2 Text) and discuss this result in more detail in the results section on p.17 and on p.18.

*Given that the mediation effect is strong, the direct effect of childhood environmental adversity on cooperation is to be interpreted with caution. Our analyses show that it is indeed a small and unstable effect, which reverses in the dataset that includes imputed data (UnStd c = 0.04 (0.02), z = 2.23, p < 0.05, Std c = 0.13). To confirm that this coefficient instability can be attributed to the presence of a mediator capturing a large part of the effect, we ran an additional model without the mediating variable (i.e., reproduction-maintenance trade-off). This analysis revealed that adverse childhood environments are indeed associated with decreased cooperation (UnStd c = -0.07 (0.01), z = -7.09, p < 0.001, Std c = -0.21).*

*The other regression coefficients are larger and more stable across analyses and datasets (i.e., the dataset implementing listwise deletions and the imputed dataset). This is the case for the regression between childhood environmental adversity and the reproduction-maintenance trade-off (listwise deletions UnStd c = 0.08 (0.01), z = 10.46, p < 0.001, Std c = 0.29; imputed data UnStd c = 0.07 (0.01), z = 6.92, p < 0.001, Std c = 0.39), for the regression between the reproduction-maintenance trade-off and cooperation (listwise deletions UnStd c = -1.07 (0.10), z = -10.87, p < 0.001, Std c = -0.73; imputed data UnStd c = -1.50 (0.34), z = -4.42, p < 0.001, Std c = -0.88), as well as for the mediation effect (listwise deletions indirect effect: UnStd c = -0.0027 (0.0004), bootstrapped ci lower = -0.0035, bootstrapped ci upper = -0.0020, z = -6.92, p < 0.001; imputed data indirect effect: UnStd c = -0.0023 (0.0004), bootstrapped ci lower = -0.0030, bootstrapped ci upper = -0.0016, z = -6.64, p < 0.001). Overall, these results converge on the idea that the relationship between childhood environmental adversity and cooperation is largely conditional on the reproduction-maintenance trade-off.*

*282-7 and S1 Text. Discussion of the effect of the remaining independent variable, parental education, is missing. Is this because of the standardisation procedure?*

That is correct. Childhood environmental adversity was modeled as a formative latent variable. These variables need to be scaled for identification purposes by fixing the coefficient of one of the causal indicators [16]. Childhood environmental adversity was scaled by setting the path from “parental education” to 1. Therefore, we do not discuss “parental education” in the results section.

*291. There seem to be words missing at the end of this line.*

We do not believe so.

*294. State what the ‘expected way’ is.*

We have changed this.

*303 & 444. ‘bold arrow’ would probably be better as ‘continuous arrow’, in contrast to the dotted arrows. Certainly in the typescript one cannot tell bold from normal lines.*

We have changed this.

*364-7. Excluding respondents without children. Further explanation would be useful here since the variable ‘only relevant for respondents with children’ (365-6), age at first birth, is not used in this second study (but is in the first). So were childless respondents excluded so that the two studies would be more similar in their respondent characteristics?*

The reason that people without children were excluded from the World Values Survey was because not having children now does not mean that you don’t want children in the future. Furthermore, removing these respondents makes it consistent with the first study (EVS), where we also removed respondents without children. We have added these arguments to the revised manuscript on p.19 and on p.20.

*390. Insert ‘respectively’ after ‘study 1’?*

We have done this.

*480. ‘whether people cooperate less under harsher conditions . . .’?*

We have changed this.

*517. ‘administrating’ should read ‘administering’.*

We have changed this.

*531. Delete ‘shocks’?*

We have done this.

*537-9. Either ‘correlates’ should read ‘correlated’ if the correlation was within the study or a reference should be given if it was a correlation with another study.*

We have given references [17,18].

*560 ‘Their model’ would read better as ‘The model’.*

We have changed this.

*830. Add journal name.*

We have done this.

**REVIEWER #2**

*Reviewer #2: This manuscript aims to build on previous research showing a negative association between “environmental harshness” and cooperation in contemporary human societies. Specifically, the authors argue that this association is mediated psychologically by a “present bias” (or future discounting) and is embodied biologically by altering the tradeoff between reproduction and maintenance in favor of reproduction. The manuscript tests this hypothesis through analysis of data from two large, cross-cultural studies: the European Values Study (EVS) and the World Values Survey (WVS).

Overall, the manuscript is clearly written and accessible, both to specialists working in this area as well as a more general scholarly audience. The theoretical and empirical motivations are linked directly to the hypotheses. The steps used to produce the data samples for analysis and the methods of analysis are both well documented. The results of the analysis are linked back to the hypotheses, and the discussion includes a helpful review of some limitations of the study and unresolved theoretical debates.

There are several additional strengths of the research featured in the manuscript. The use of large, cross-cultural surveys presents opportunities to test associations between “environmental harshness” and cooperation in a variety of contexts with measures that arise from a common survey methodology, which should enhance the quality of comparisons. The large sample sizes in these data sets also allow the authors to develop their models using one half of the sample, then validate these models with the other half. The analysis explores direct associations between “environmental harshness” and cooperation, as well as mediations via the trade-off between reproduction and maintenance, which unites two strands of research on environmental harshness that are often pursued separately. The discussion of analytic methods and interpretation of results are also sufficiently nuanced to take into account both the strengths and weaknesses of the data used in the study, which helps clarify the contributions of this research as well as opportunities for future studies to build on them. I also appreciated the choice to focus on “collective action” variables from these particular data sets, rather than moral attitudes about cooperation, which have been the focus of previous research.

Along this these strengths, there are several areas where the manuscript could be improved. I outline these below:

1. The manuscript uses the term “environmental harshness” to refer to a broad range of conditions, defined by the “rate at which external morbidity and mortality causes disability & death in a population” (p.3). The authors go on to explain that this often manifests in contemporary human populations as “economic deprivation and resource scarcity.” I found the use of the term “environmental harshness” to be somewhat confusing in this regard, as I was expecting the research to focus more on ecological conditions rather than social conditions tied to “harshness.” Considering the countries included in these data sets, the real-world challenges reflected in the variables used to operationalize the concept of “harshness” are more directly tied to social conditions and dynamics than they are to ecological conditions and dynamics. The manuscript is fairly clear in how it defines the term “environmental harshness,” so this is not a major concern, but I do wonder if it would be closer to the spirit of the analysis to replace this term with something like “economic deprivation and scarcity,” which probably better reflects the specific measures used in the study and the social contexts where these surveys were conducted.*

We fully agree with the reviewer that “environmental harshness” intuitively might be more linked to ecological conditions than to social conditions. We therefore changed the term “environmental harshness” to the more neutral term “environmental adversity”. For the childhood environment we now use the term “childhood environmental adversity” and for the current environment we now use the term “current environmental adversity”. The definition of “environmental adversity” is now given op p.3 of the revised manuscript and the new terms are inserted throughout the revised manuscript.

*Environmental adversity is often conceptualized as the product of two different dimensions: harshness (i.e., externally caused levels of morbidity-mortality that an individual cannot control) and unpredictability (i.e., spatial-temporal variation in harshness) [1].*

*2. Related to the concern about the definition of “environmental harshness” described above, I felt the manuscript could do more to consider recent research on the evolution of cooperation that shows a positive association between environmental harshness and cooperation. For example, several papers by Smaldino and colleagues (2013a; 2013b; 2013c) use agent-based models to show that increasing environmental harshness leads to increasing cooperation, as does research by Aktipis, Cronk and colleagues (2011; 2015; 2016; 2019) These positive associations run in the opposite direction as most of the research cited in the manuscript, which is presented as the current consensus from an empirical standpoint. The studies showing positive associations also focus more directly on ecological conditions linked to “harshness,” so perhaps this is one reason they diverge from those cited in this manuscript, which share the focus on economic deprivation and scarcity. Regardless, I would like to see this manuscript engage more directly with this work showing positive associations between environmental harshness and cooperation, both in terms of its theoretical framework and interpretation of its results.*

We thank the reviewer for highlighting this issue. The question whether environmental adversity is associated with decreased or increased cooperation is still debated. We are aware of the studies that found no significant effect between environmental adversity and cooperation or found the opposite effect [19–24].

The reason for the inconsistent findings is unknown. Whether this is due to adversity being linked to ecological conditions or socio-economical conditions is a good suggestion. Lazarus [1] also suggests that in general, environmental adversity is associated with increased cooperation, except when adversity is measured in terms of socioeconomic position, then the pattern is reversed.

Elsewhere, we have argued that the inconsistent findings in the literature could be due to the way cooperation is measured. In theory, the relationship between adversity and cooperation could go either way [20,22,25]. The existing empirical evidence is currently inconclusive. On the one hand, when cooperation is measured via self-reported questionnaires or in field experiments, the pattern of results is rather consistent and suggestive of an association between adverse environments and decreased cooperation. On the other hand, when cooperation is measured using behavioral experiments (i.e., economic games and actual behavior in various settings under scrutiny of the experimenter), the results are much more mixed.

Because we measure cooperation via self-reported questionnaires, we expected that we would find an association between adverse environments and decreased cooperation, which we in fact did. We have added the arguments for the inconsistent findings in the introduction of the revised manuscript on p.3 and p.4.

*However, whether cooperation is decreased or increased in adverse environments is still debated. In theory, the relationship between environmental adversity and cooperation could go either way [30]. On the one hand, adverse environments might correlate with increased cooperation as a means to mitigate direct survival threats, such as pooling material resources to reduce the impact of external events like famine [31,32]. On the other hand, adverse environments might correlate with decreased cooperation because adversity constrains people to focus on trying to meet their individual needs [30].*

*The existing empirical evidence is currently inconclusive. When cooperation is measured via self-reported questionnaires or in field experiments, studies usually report an association between adverse environments and decreased cooperation. Notably, Korndörfer et al. [33] and Schmukle et al. [34] found a negative correlation between lower social class and cooperative behaviors (e.g., donating, volunteering, helping) using questionnaire data from large and representative international samples (average N over 9 studies = 20.243). Using field experiments, Andreoni et al. [35] and Nettle et al. [36] also found that lower social class correlates with decreased cooperation.*

*When cooperation is measured using behavioral experiments (i.e., economic games and actual behavior in various settings under scrutiny of the experimenter), the results are much more mixed. Some studies report an association between adverse environments and decreased cooperation [33,34,36–38], but other studies find the opposite pattern [39–41] or no effect at all [42,43].*

*The reason why these behavioral experiments give mixed results is unclear, but several explanations have been put forward. First, some of these studies have small sample sizes, which may explain, for example, why Piff et al. [41], who found a positive relation between environmental adversity and cooperation, was not replicated in a high-powered pre-registered study [42]. Second, these studies usually only use one economic game, which may lower their generalizability [44]. In accordance with this idea, McAuliffe et al. [45] found that single economic games do not correlate with real-life cooperation, but a general factor based on several economic games does. Finally, there is increasing evidence that economic games lack ecological validity [46].*

*3. The authors suggest one of the manuscript’s primary contributions to research in this field is to test the idea that the association between environmental harshness and cooperation is mediated by a present bias at the psychological level that is in turn embodied at the biological level in terms of reproductive strategies. This is an interesting idea that builds on previous research on variation in human life history strategies in relation to environmental harshness, but there are some limitations in the analysis featured in this manuscript that I think should be discussed. First, the analysis does not examine present bias or other psychological mechanisms directly. Instead, it infers these from specific strategies for reproduction (e.g. early reproduction, more children) and cooperation (e.g. fewer contributions to collective action). These inferences are generally logical, but there may be alternative explanations that don’t involve present bias at the psychological level. For example, research by Amir and colleagues on this topic (2019) has suggested that risk management is an alternative explanation, which is not cited nor discussed in the manuscript. Considering the possibility that reproductive and cooperative strategies may also be shaped by risk management, I feel the authors should address this alternative explanation (and the empirical research supporting it) in their manuscript. Indeed, Amir et al (2018) have empirical evidence that environmental harshness is positively associated with cooperation, connecting this issue to the previous one. Perhaps there are even variables in the EVS and WVS that could be used to test this alternate hypothesis. Also, it’s worth noting that some of the work by this research team also provides support for some of the associations presented in this manuscript (Amir et al 2016).*

The reviewer is right in pointing out that we did not examine present bias or other psychological mechanisms directly. Furthermore, through what mechanism adversity is related to cooperation in still unclear and several explanations have been put forward, including risk management.

Amir et al. [19] put forward an uncertainty management perspective, where adverse environments are associated with risk averse, present-oriented and more cooperative preferences. Although, they do not specifically test whether risk is a mediator between environmental adversity and cooperation. Even in the uncertainty management perspective proposed by Amir et al. [19], adverse environments are associated with a present-orientation, which is in line with the framework of our study. Amir et al. [19] see cooperation as a form of risk pooling which would reduce uncertainty. Therefore, in adverse and uncertain environments, this would be associated with increased cooperation.

We fully agree that in adverse environments people will want to reduce uncertainty, but we are not entirely convinced that the primary means to achieve this is increased cooperation. One possibility is that this might be the case in specific contexts, for example, where population size is low and where the likelihood of interacting with a third party outside the kinship community is small. However, the pattern might be reversed in places where the population size is larger and where the likelihood of interacting with non-kin increases, because the likelihood of being cheated also increases as a result [26–28], and this risk might further increase in adverse conditions. In these environments, trusting other people less and cooperating less can be viewed as a means to reduce uncertainty, a view which is in contradiction with Amir et al.’s findings [19], but which fits a large set of work in the social sciences [29–31] as well as our own results.

The negative relationship found by Amir et al. [19] between environmental adversity and cooperation might also be due to the way cooperation is measured (see comment #2). We do not have specific hypotheses about the effect of adversity on risk-orientation (in accordance with the delay discounting account, see Amir et al. [19]), although this is an interesting topic for further research, we chose not to include it in our study.

We used the reproduction-maintenance trade-off as a possible mediator between the relationship of environmental adversity and cooperation, but we fully agree that there could be other possible mediators, including risk management. In accordance with the reviewer’s suggestion, we include risk management as an alternative explanation in the discussion section of the revised manuscript on p.29 and on p.30.

*Another mechanism by which environmental adversity can have an effect on cooperation is risk management. For example, Amir et al. [39] put forward an uncertainty management perspective, where adverse environments are associated with risk averse, present-oriented and more cooperative preferences. Although, they do not specifically test whether risk is a mediator between environmental adversity and cooperation. Even in the uncertainty management perspective proposed by Amir et al. [39], adverse environments are associated with a present-orientation, which is in line with the framework of our study. Amir et al. [39] see cooperation as a form of risk pooling which would reduce uncertainty. Therefore, in adverse and uncertain environments, this would be associated with increased cooperation. We fully agree that in adverse environments people will want to reduce uncertainty, but we are not entirely convinced that the primary means to achieve this is increased cooperation. One possibility is that this might be the case in specific contexts, for example, where population size is low and where the likelihood of interacting with a third party outside the kinship community is small. However, the pattern might be reversed in places where the population size is larger and where the likelihood of interacting with non-kin increases, because the likelihood of being cheated also increases as a result [130–132], and this risk might further increase in adverse conditions. In these environments, trusting other people less and cooperating less can be viewed as a means to reduce uncertainty, a view which is in contradiction with Amir et al.’s findings [39], but which fits a large set of work in the social sciences [81,83,133] as well as our own results. We think that risk management strategies in the contest of social behaviors are an interesting topic for further research.*

*4. There are several missing details about the EVS and WVS data sets that are important for interpreting the results featured in the manuscript. First, the manuscript mentions the number of countries included in each data set, but does not explain which countries are involved. This is more important for the WVS than the EVS, because part of the justification for including analysis from the WVS was to expand the sample of countries considered beyond Europe. I recommend the authors list these countries included in the samples used for analysis for both data sets, either in the main text or in a table included in the main text, perhaps along with descriptive stats at the country level for each of the main variables. Second, the manuscript should engage with or at least acknowledge the extent of variation in social and ecological contexts that are and are not reflected in these samples. Does environmental harshness (or economic deprivation and scarcity) manifest in similar ways in all countries, or are there variations that should be accounted for? More details along these lines would help develop some key parts of the manuscript where this issue arises. For example, in the discussion, the manuscript notes the fact that “number of children” did not substantially contribute to the construct of the reproduction-maintenance tradeoff in the EVS, but it did in the WVS (p.22). The discussion suggests this may be due to the use of contraceptives and other “cultural factors.” This would be a good opportunity to provide more details about the countries included, specifically variation in demographic profiles and other factors linked to reproduction, as well as factors linked to the measures of environmental harshness and collective action. Finally, the modeling strategy doesn’t appear to account in any way for differences at the country level when estimating the associations among the key variables. A multilevel modeling approach might help clarify the extent that the associations reported in the manuscript are general across all countries.*

We thank the reviewer for these suggestions. We have included a table with descriptive statistics for each country in the Supporting Information (S1 Table for the EVS and S5 Table for the WVS).

Furthermore, we chose to perform our analyses on the individual respondents and not on the country level for several reasons. First, we do not have enough data per country (EVS: range 4-571; WVS: range 157-908) to run our multivariate models on each of them. Second, an infinite number of aggregated factors might explain the individual level data between environmental adversity, the reproduction-maintenance trade-off and cooperation. For example, countries’ geographical vicinity, linguistic vicinity, population density, GDP per capita, history of pathogen load, education rate, political system or any other forms of cultural norms. The final choice is necessarily arbitrary even when it is theoretically informed. Therefore, to avoid this pitfall we decided to give our priority to the individual level of description.

*5. The manuscript’s presentation of results and discussion focuses mostly on the statistical significance of the documented associations, but there is little discussion of effect sizes, aside from a brief mention that the reported effect sizes are generally small (p.14), as well as how the effect sizes for the direct association between environmental harshness and collective action decrease when accounting for mediation via reproductive strategies (p.15). This left me wondering about the substantive significance of these associations in relation to observed variation in the key variables. The bivariate correlation coefficients appear to suggest relatively little co-variance in the two composite collective action measures (volunteering and political action) and the variables representing the two predictor constructs. The large sample sizes used in this study raise the possibility that the statistically significant associations reported are due primarily to the high statistical power enabled by the large sample sizes used in this analysis, rather than effect sizes. More detailed discussion of effect sizes and substantive significance would help balance the interpretation of full significance of these associations.*

We fully agree with the reviewer that the large sample sizes of our studies make the reporting of the p-values less informative. We now also report under the z label the value of the Wald test – the ratio between the parameter value and its standard error – as an indicator of the effect size. In accordance with the reviewer’s suggestion, we discuss the effect sizes in the results section of our revised manuscript on p.24.

*Despite the fact that the association pattern reported above is identical to the one found with the European Values Study (EVS) data, one should note that the effects are smaller (by comparing the z-values). This is the case for the regression between environmental adversity and cooperation (EVS UnStd c = -0.03 (0.01), z = -3.50, p < 0.001, Std c = -0.08; WVS UnStd c = 0.12 (0.11), z = 1.12, p = 0.26, Std c = 0.25), between environmental adversity and the reproduction-maintenance trade-off (EVS UnStd c = 0.08 (0.01), z = 10.46, p < 0.001, Std c = 0.29; WVS UnStd c = 0.42 (0.10), z = 4.36, p < 0.001, Std c = 0.70), between the reproduction-maintenance trade-off and cooperation (EVS UnStd c = -1.07 (0.10), z = -10.87, p < 0.001, Std c = -0.73; WVS UnStd c = -0.48 (0.19), z = -2.49, p < 0.05, Std c = -0.60) as well as for the mediation effect (EVS indirect effect: UnStd c = -0.0027 (0.0004), bootstrapped ci lower = -0.0035, bootstrapped ci upper = -0.0020, z = -6.92, p < 0.001; WVS indirect effect: UnStd c = -0.003 (0.001), bootstrapped ci lower = -0.004, bootstrapped ci upper = -0.001, z = -2.67, p < 0.01).*

*6. Finally, the Discussion section ends rather abruptly with a discussion of limitations and unresolved issues. I suggest the authors consider including at least a short paragraph or two to return to the main contributions of this work and perhaps suggest some important directions for further research in this area, which would help return to the general motivations of this work.*

We thank the reviewer for this helpful suggestion. We have included a final paragraph on p.30 of the revised manuscript.

*Whether cooperation is decreased or increased in adverse environments is still debated. The present study contributes to this debate by reporting two pre-registered studies applying structural equation models to test this relationship on large-scale datasets (the European Values Study and the World Values Survey). The results show that adverse environments in childhood and adulthood are associated with decreased investment in adult cooperation. Furthermore, the channel through which this relationship arises is also unclear. We proposed that this relationship is mediated by a present bias at the psychological level, which is embodied in the reproduction-maintenance trade-off at the biological level. We indeed found that the relationship between environmental adversity and cooperation is mediated by individuals’ reproduction-maintenance trade-off.*

**REFERENCES**

1.	Lazarus J. Cooperation in adversity: An evolutionary approach. Global Discourse. 2017 Oct 2;7(4):571–98. doi: 10.1080/23269995.2017.1402426.

2.	West SA, El Mouden C, Gardner A. Sixteen common misconceptions about the evolution of cooperation in humans. Evolution and Human Behavior. 2011 Jul 1;32(4):231–62. doi: 10.1016/j.cub.2007.06.004.

3.	West SA, Griffin AS, Gardner A. Evolutionary explanations for cooperation. Current Biology. 2007 Aug 21;17(16):R661–72. doi: 10.1016/j.evolhumbehav.2010.08.001.

4.	Hamilton WD. The genetical evolution of social behaviour. II. Journal of Theoretical Biology. 1964 Jul 1;7(1):17–52. doi: 10.1016/0022-5193(64)90039-6.

5.	Carpenter J, Myers CK. Why volunteer? Evidence on the role of altruism, image, and incentives. Journal of Public Economics. 2010 Dec 1;94(11):911–20. doi: 10.1016/j.jpubeco.2010.07.007

6.	Menchik PL, Weisbrod BA. Volunteer labor supply. Journal of Public Economics. 1987 Mar 1;32(2):159–83. doi: 10.1016/0047-2727(87)90010-7.

7.	Ziemek S. Economic analysis of volunteers’ motivations—A cross-country study. The Journal of Socio-Economics. 2006 Jun 1;35(3):532–55. doi: 10.1016/j.socec.2005.11.064.

8.	Ali SN, Lin C. Why people vote: Ethical motives and social incentives. American Economic Journal: Microeconomics. 2013 May;5(2):73–98. doi: 10.1257/mic.5.2.73.

9.	Gerber AS, Huber GA, Doherty D, Dowling CM. Why people vote: Estimating the social returns to voting. British Journal of Political Science. 2016 Apr;46(2):241–64. doi: 10.1017/S0007123414000271.

10.	Pepper GV, Nettle D. The behavioural constellation of deprivation: Causes and consequences. Behavioral and Brain Sciences. 2017 ed;40:1–72. doi: 10.1017/S0140525X1600234X.

11.	Price ME, Cosmides L, Tooby J. Punitive sentiment as an anti-free rider psychological device. Evolution and Human Behavior. 2002 May 1;23(3):203–31. doi: 10.1016/S1090-5138(01)00093-9.

12.	Delton AW, Cosmides L, Guemo M, Robertson TE, Tooby J. The psychosemantics of free riding: Dissecting the architecture of a moral concept. Journal of Personality and Social Psychology. 2012;102(6):1252–70. doi: 10.1037/a0027026.

13.	Côté S, House J, Willer R. High economic inequality leads higher-income individuals to be less generous. Proceedings of the National Academy of Sciences. 2015;112(52):15838–15843. doi: 10.1073/pnas.1511536112.

14.	Schmukle SC, Korndörfer M, Egloff B. No evidence that economic inequality moderates the effect of income on generosity. PNAS. 2019 May 14;116(20):9790–5. doi: 10.1073/pnas.1807942116.

15.	Lim D, DeSteno D. Suffering and compassion: The links among adverse life experiences, empathy, compassion, and prosocial behavior. Emotion. 2016;16(2):175–82. doi: 10.1037/emo0000144.

16.	Mell H, Safra L, Algan Y, Baumard N, Chevallier C. Childhood environmental harshness predicts coordinated health and reproductive strategies: A cross-sectional study of a nationally representative sample from France. Evolution and Human Behavior. 2018 Jan 1;39(1):1–8. doi: 10.1016/j.evolhumbehav.2017.08.006.

17.	Kagel J, McGee P. Personality and cooperation in finitely repeated prisoner’s dilemma games. Economics Letters. 2014 Aug 1;124(2):274–7. doi: 10.1016/j.econlet.2014.05.034.

18.	Koole SL, Jager W, van den Berg AE, Vlek CAJ, Hofstee WKB. On the social nature of personality: Effects of extraversion, agreeableness, and feedback about collective resource use on cooperation in a resource dilemma. Pers Soc Psychol Bull. 2001 Mar 1;27(3):289–301. doi: 10.1177/0146167201273003.

19.	Amir D, Jordan MR, Rand DG. An uncertainty management perspective on long-run impacts of adversity: The influence of childhood socioeconomic status on risk, time, and social preferences. Journal of Experimental Social Psychology. 2018 Nov 1;79:217–26. doi: 10.1016/j.jesp.2018.07.014.

20.	Aktipis CA, Cronk L, de Aguiar R. Risk-pooling and herd survival: An agent-based model of a Maasai gift-giving system. Hum Ecol. 2011 Apr 1;39(2):131–40. doi: 10.1007/s10745-010-9364-9.

21.	Piff PK, Kraus MW, Côté S, Cheng BH, Keltner D. Having less, giving more: The influence of social class on prosocial behavior. Journal of Personality and Social Psychology. 2010;99(5):771–84. doi: 10.1037/a0020092.

22.	Smaldino PE, Schank JC, McElreath R. Increased costs of cooperation help cooperators in the long run. The American Naturalist. 2013 Apr 1;181(4):451–63. doi: 10.1086/669615.

23.	Stamos A, Lange F, Huang S, Dewitte S. Having less, giving more? Two preregistered replications of the relationship between social class and prosocial behavior. Journal of Research in Personality. 2020 Feb 1;84:103902. doi: 10.1016/j.jrp.2019.103902.

24.	Wu J, Balliet D, Tybur JM, Arai S, Van Lange PAM, Yamagishi T. Life history strategy and human cooperation in economic games. Evolution and Human Behavior. 2017 Jul 1;38(4):496–505. doi: 10.1016/j.evolhumbehav.2017.03.002.

25.	Nettle D. Tyneside Neighbourhoods: Deprivation, Social Life and Social Behaviour in One British City. Open Book Publishers; 2015. doi: 10.11647/OBP.0084.

26.	Boyd R, Richerson PJ. The evolution of reciprocity in sizable groups. Journal of Theoretical Biology. 1988 Jun 7;132(3):337–56. doi: 10.1016/S0022-5193(88)80219-4.

27.	Nowak MA, Sigmund K. Evolution of indirect reciprocity by image scoring. Nature. 1998 Jun;393(6685):573–7. doi: 10.1038/31225.

28.	Suzuki S, Akiyama E. Reputation and the evolution of cooperation in sizable groups. Proceedings of the Royal Society B: Biological Sciences. 2005 Jul 7;272(1570):1373–7. doi: 10.1098/rspb.2005.3072.

29.	Guiso L, Sapienza P, Zingales L. Does culture affect economic outcomes? Journal of Economic Perspectives. 2006 Jun;20(2):23–48. doi: 10.1257/jep.20.2.23.

30.	Inglehart R. Trust, well-being and democracy. Democracy and trust. 1999;88.

31.	Ortiz-Ospina E, Roser M. Trust. Published online at OurWorldInData.org; 2019. Available from: https://ourworldindata.org/trust
