# Supplementary material for: Environmental adversity is associated with lower investment in collective actions
Source: PLoS One. 2020 Jul 30;15(7):e0236715. doi: 10.1371/journal.pone.0236715 (PMC7392252; doi:10.1371/journal.pone.0236715)
Supplement: Attachment — Submitted filename: Response to Reviewers.docx [file pone.0236715.s017.docx]

***Reviewer #1***

*My replies to the authors’ responses are headed by the numbered points from my first review (or by line numbers in the first reviewed paper, following ‘pp’) in square brackets, followed by page number in the Track Changes version of the revised paper. (In their response the authors use the page numbers of the revised clean version but in checking the revision the Track Changes version is preferable.)

I appreciate the authors’ constructive engagement with my comments and their detailed and considered responses. None of my comments are major enough to require another look at the paper.

Looking at this again I see that figures 1 and 3 are redundant and could be removed; figures 2 and 4 repeat them, respectively, while adding the statistics.*

We removed the two figures. *[3] 98-103. Definition of cooperation. Now that the authors provide a source (West et al., references 61, 62) for their definition, and explain it, this point is satisfactorily resolved.

[3, particularly 3a, b, e] 136-7, 236-49. The authors are now explicit about the potential delayed benefits of volunteering and political action (with supporting references), and have defined ‘collective action’. So this is OK.

[4] ‘the discrepant findings in the literature about the effect of environmental harshness on prosociality (the authors refer to this at 42-6) may be partly a function of the literature not distinguishing carefully enough between different types of social interaction’. This is a general point that I invited the authors to engage with but it is not essential for their paper that they do so. Although they did not take up this point they did add an interesting discussion (54-83) of a second point I raised here that, ‘. . . it is also important to take account of the methodology by which these results have been obtained, from behavioural observations and experiments to self-report, in both the lab and the real world, since they each have their own psychological influences.’

[pp 255-9] 392-413. The new analysis and comparisons between existing analyses in response to my comment are a useful addition to the paper. However, the authors should state at 396-9 whether the new analysis is (a) on the replication or the discovery sample, and (b) on the listwise deletion or imputed data set.*

The analysis was performed on the replication sample with imputed data. We added this information on p.17 of the revised manuscript.

*All other comments have been answered satisfactorily.

Comments on new text & new supplementary material

57, 60. ‘correlate’ not the best word here since causation is proposed.*

Although causation is proposed, the studies where this statement is based on do not test causality specifically. Therefore we changed “correlate” with “associate”.

*310-12 & S1 Text. Largely ‘consistent with the replication sample’ yes, but point out that there are two additional significant associations with Childhood adversity in the discovery sample and name them.*

We added “parents had problems makings ends meet” and “death of mother”.

*393, 399, 405, 412, 560, 564. To stick to the variable name in the studies, as elsewhere in the paper, ‘cooperation’ should read ‘collective action’.*

We have changed this.

*456-7. Please explain the logic of ‘because not having children now does not mean that you don’t want children in the future’.*

The reason is that people who do not have children now fall into three categories: (1) people who do not have children yet but will do so in the future, (2) people who do not have children yet and will choose not to do so in the future and (3) people who do not have children yet but would like to and can’t. Since we have no way of distinguishing these three groups, we focused on the people who do have children.

*634. ‘linear’ should read ‘monotonic’.*

We have changed this.

*707. ‘contest’ should read ‘context’; ‘are’ should read ‘is’.*

We have changed this.

*687-707. With reference to this discussion on uncertainty the authors might be interested in the following theoretical paper, co-authored by myself, but there is no need to cite it here: Andras, P., Lazarus, J., & Roberts, G. (2007). Environmental adversity and uncertainty favour cooperation. BMC Evolutionary Biology, 7, 240. doi: 10.1186/1471-2148-7-240.

Reference 1 has a subtitle, to be added: ‘The impact of harsh versus unpredictable environments on the evolution and development of life history strategies’.*

We have added this.

*References 61 & 62. The journal names in the doi of each reference have been switched.*

We have changed this.

*Reference 133 is perhaps incomplete.*

We do not believe so.

*S1 Text (and check other text and tables for same comment). As I’m sure the authors know, p values aren’t actually zero, statistics packages merely print them this way; ‘0.00’ should read ‘<0.005’.*

We have changed the *p*-values from “0.00” to “<0.001” in all the tables and text.

***Reviewer #2***

*In addition to the strengths of the initial submission, my review highlighted six issues for improvement. I have read the authors’ reply to each issue and reviewed the relevant changes made to the revised manuscript. I am satisfied with the responses and revisions and support publication of this manuscript. I offer more specific replies on each point below:

1. Use of the term “environmental harshness.”
- The authors have replaced the term “environmental harshness” with “environmental adversity.” I support this change and feel it helps clarify the specific social and environmental conditions that are the focus of this manuscript in relation to variation in reproductive strategies and cooperation.

2. Positive or negative association between environmental adversity and cooperation?
- The revised manuscript has an expanded review of published literature on this question and effectively highlights studies that find both positive and negative associations. I particularly like the way the authors explore possible methodological factors that might help explain these diverging results and link this idea to the results featured in their manuscript. My only suggestions for improving this aspect of the manuscript further would be: a) to reflect on and explore possible differences between self-reported measures of cooperation and behavioral measures (especially via economic experiments or “games”) and b) to add a note explaining that several of the studies cited as support for a positive association between environmental adversity and cooperation use agent-based models as a methodology, which is distinct in important ways from both self-reported and behavioral economics-based measures.*

We thank the reviewer for these suggestions. We have added several differences between self-reported measures of cooperation and economic games on p.3 and p.4 of the revised manuscript. Furthermore, point b which the reviewer suggests is added on p.4 of the revised manuscript.

*3. Alternative explanations/mechanisms underlying associations between environmental adversity and present bias.
- The revised manuscript features an extended discussion of alternative explanations/mechanisms, including uncertainty management. I appreciate the way the authors consider these alternatives and discuss how the results in this manuscript do and do not align with them.*

*4. More information on EVS and WVS data sets.
- The supplementary files associated with the manuscript now provide more detailed data about the countries included in the EVS and WVS data sets, as well as descriptive statistics for the variables used in analysis. I did notice one possible issue (hopefully minor) in Table S1 for the EVS, the range for the “Age1stBirth” variable goes from 12-83. My understanding is that this variable represents the age when each person responding to the survey first became a parent. Does this mean that the oldest parent in the data set was 83 years old when their first child was born? This would be very old for a male (but not unprecedented) and would, as far as I can tell, be a world record for a female. If the authors have not already looked into this, it might be good to double check the extreme old/young values for this variable in relation to other variables (gender, age) to make sure there are no errors in this important variable.*

The reviewer is correct in that the variable “age at first birth” represents the age of the respondents when they first became a parent. In our sample, over 99% of the respondents had their first child between the ages of 16 and 43. However, less than 1% is younger or older when they had their first child. The number of female respondents who stated that they had their first child after the age of 50 was 14 out of 17.087. We suspect that some of these data points are in fact entry errors. However, we kept the full sample for two reasons: first, it is impossible to tease apart entry errors from true late first births and second, very few individuals reported to have had their first birth at a later age (only 14 women out of 17.087). To ensure that the inclusion of these 14 participants did not alter our results we ran the model again without these participants and found the same overall pattern. We have included this information on p.18 of the revised manuscript.

*5. Effect sizes for SEMs.
- The authors have added some additional information about effect sizes for the associations reported in the SEMs and include some discussion of effect sizes in their assessment of the relationships between environmental adversity, reproduction, and cooperation. The manuscript’s conclusions continue to emphasize results of statistical significance tests the direction of associations, but given the large sample sizes, I still feel the discussion of effect size could be expanded to establish links to the substantive significance of these associations. Perhaps this could be done by linking the estimated coefficients to units of measurement for each variable, then discussing what would constitute a meaningful change in levels of cooperation and how large a change in environmental adversity/reproductive strategy is required to produce this change, according to the SEMs. Perhaps the authors may find this recent article useful in this regard:*

*Brenna Gomer, Ge Jiang & Ke-Hai Yuan (2019) New Effect Size Measures for Structural Equation Modeling, Structural Equation Modeling: A Multidisciplinary Journal, 26:3, 371-389, DOI: 10.1080/10705511.2018.1545231*

We thank the reviewer for this suggestion. We have read the paper but as far as we can discern, the effect sizes mentioned concern the whole model fit, not individual parameters.

Standardized regression coefficients can be used as an effect size index [1]. Standardized regression coefficients refer to how many standard deviations the dependent variable will change per a standard deviation increase in the independent variable, holding all other variables constant. Standardized regression coefficients can be interpreted similar to correlations, where <0.2 is considered a weak effect, between 0.2 and 0.5 as a moderate effect and >0.5 as a strong effect [2].

In accordance with the reviewer’s suggestion, we discuss the effect sizes (based on the standardized regression coefficients) in more detail in the discussion section of the revised manuscript on p.27.

*6. Summary paragraph.
- The revised manuscript contains a summary paragraph at the end of the Discussion section that nicely states the key contributions of this study.

I thank the authors for their considerate responses to the issues raised in my initial review.*

**REFERENCES**

1. Nieminen P, Lehtiniemi H, Vähäkangas K, Huusko A, Rautio A. Standardised regression coefficient as an effect size index in summarising findings in epidemiological studies. Epidemiology, Biostatistics and Public Health. 2013;10(4).
2. Acock AC. A gentle introduction to Stata. College Station. Texas: Stata Press; 2014.
